# Multi‐country investigation of the diversity and associated microorganisms isolated from tick species from domestic animals, wildlife and vegetation in selected african countries

**DOI:** 10.1007/s10493-021-00598-3

**Published:** 2021-03-01

**Authors:** Emanuela Olivieri, Edward Kariuki, Anna Maria Floriano, Michele Castelli, Yohannes Mulatu Tafesse, Giulia Magoga, Bersissa Kumsa, Matteo Montagna, Davide Sassera

**Affiliations:** 1 Department of Biology and Biotechnology, University of Pavia, via Ferrata 9, 27100 Pavia, Italy; 2 Department of Veterinary Service, Wildlife Service, Nairobi, Kenya; 3 Dipartimento di Scienze Agrarie e Agroambientali, Università degli Studi di Milano, via Celoria 2, 20133 Milan, Italy; 4 Department of Parasitology, College of Veterinary Medicine, Addis Ababa University, P.O Box 34, Bishoftu, Ethiopia; 5 BAT Center - Interuniversity Center for Studies on Bioinspired Agro-Environmental Technology, University of Napoli ‘Federico II’, 80138 Portici, Italy

**Keywords:** Ticks, Endosymbionts, Tick‐borne pathogens, Co‐infection, Africa

## Abstract

In many areas of Africa, recent studies highlighted the great impact of ticks on animal and human health throughout the continent. On the other hand, very limited information on the bacterial endosymbionts of the African ticks and their pattern of co-infections with other bacteria are found in literature, notwithstanding their pivotal role in tick survival and vector efficiency. Thus, we investigated the distribution of selected pathogenic and symbiotic bacteria in hard ticks collected from wild, domestic animals and from vegetation in various ecological zones in Africa and their co-occurrence in the same tick host. Overall, 339 hard ticks were morphologically identified as belonging to the genera *Amblyomma*, *Dermacentor*, *Hyalomma*, *Haemaphysalis, Ixodes* and *Rhipicephalus*. Molecular screening provided information on pathogens circulation in Africa, detecting spotted fever group rickettsiae, *Anaplasma* spp., *Ehrlichia ruminantium*, *Borrelia garinii*, *Babesia* spp., *Theileria* spp. and *Coxiella burnetii*. Furthermore, our work provides insights on the African scenario of tick-symbiont associations, revealing the presence of *Coxiella*, *Francisella* and *Midichloria* across multiple tick populations. *Coxiella* endosymbionts were the most prevalent microorganisms, and that with the broadest spectrum of hosts, being detected in 16 tick species. *Francisella* was highly prevalent among the *Hyalomma* species tested and correlated negatively with the presence of *Coxiella*, showing a potential competitive interaction. Interestingly, we detected a positive association of *Francisella* with *Rickettsia* in specimens of *Hy. rufipe*s, suggesting a synergistic interaction between them. Finally, *Midichloria* was the most prevalent symbiont in *Rhipicephalus sanguineus* sensu lato from Egypt.

**Supplementary Information:**

The online version contains supplementary material available at 10.1007/s10493-021-00598-3.

## Introduction

Over the last few decades, a growing number of studies have focused on exploring the composition of microbial communities harboured by blood-feeding arthropods, such as ticks (Acari: Ixodidae), reporting a mixture of commensal, mutualistic and pathogenic microorganisms (Andreotti et al. 2011; Narasimhan and Fikrig 2015; Bonnet et al. 2017; Duron et al. 2017). As a result of these efforts, a number of new tick-borne pathogens (TBPs) and new microbial associations have been described (Vayssier-Taussat et al. 2013; Greay et al. 2018). Many of these microorganisms can coexist simultaneously within the same host and synergistic or antagonistic interactions have been hypothesized (Vautrin and Vavre 2009; Moutailler et al. 2016; Díaz-Sánchez et al. 2019) and also proven in specific cases (Paddock et al. 2015; Budachetri et al. 2018).

In many areas of Africa, recent studies highlighted the great impact of ticks on animal and human health throughout the continent (Jongejan and Uilenberg 2004; Maina et al. 2014; Lorusso et al. 2016; Kamani et al. 2018; Asante et al. 2019). The local environmental conditions together with the close contact of wildlife animals with domestic animals and humans provide the opportunities for colonizing multiple niches, driving the spread of TBPs. The most common zoonotic bacteria reported in Africa are the spotted fever group (SFG) rickettsiae, mainly represented by *Rickettsia africae*, *R. aeschlimannii*, *R. conorii* and *R. massiliae* (Macaluso et al. 2003; Parola et al. 2005). The circulation of pathogens of veterinary importance have also been commonly reported, including *Ehrlichia ruminantium*, *Anaplasma marginale*, *A. phagocytophilum*, and *A. centrale*, widespread among ruminants (Bekker et al. 2002; Ikwap et al. 2010; Allsopp 2015), and piroplasms (*Babesia* spp. and *Theileria* spp.), which infect ruminants and equids (Gebrekidan et al. 2014; Hawkins et al. 2015).

Whereas studies on TBPs in Africa are flourishing, to date there is very limited information regarding the bacterial endosymbionts of the African ticks and their pattern of co-infections with other bacteria. Endosymbionts, intracellular bacteria with high prevalence and load that are generally transovarially transmitted, have been proven to be fundamental in the survival of hematophagous arthropods, ticks included, and thus warrant extensive investigation. The main bacterial endosymbionts of ticks are *Coxiella* (order *Legionellales*), *Francisella* (order *Thiotrichales*), ‘*Candidatus* Midichloria’ and *Rickettsia* (order *Rickettsiales*) (Duron et al. 2017). The most common tick endosymbiont is *Coxiella*, detected in most individuals of numerous tick species (Clay et al. 2008; Lalzar et al. 2012; Machado-Ferreira et al. 2016; Duron et al. 2017). Recent studies focused on the intricate interaction of this symbiont in ticks showed that *Coxiella* endosymbionts possess the typical hallmarks of an obligate symbiont from a physiological point of view. For example, their pronounced tropism to the host ovary is indicative of the predominantly maternal transmission (typical of bacterial intracellular symbionts), and the negative effect on the hosts physiology caused by a reduction of the symbiont load is consistent with a mutualistic role (Zhong et al. 2007; Guizzo et al. 2017; Zhang et al. 2017). Such role is thought to be the provisioning of essential nutrients. Indeed, the presence of B vitamins and cofactors biosynthesis pathways in genomes of different strains of *Coxiella* endosymbionts suggest their capability of supplementing the unbalanced blood diet of the hosts (Gottlieb et al. 2015; Smith et al. 2015). *Coxiella* is believed to be the bacterium with the oldest symbiotic association with tick hosts, but other endosymbiotic bacteria, especially *Francisella*, have been reported to have a similar role, possibly having replaced *Coxiella* in some tick species (Duron et al. 2017).

Indeed, *Francisella* endosymbionts have been commonly reported in *Coxiella-*free ticks, belonging to the genera *Dermacentor*, *Amblyomma*, *Hyalomma* and *Ornithodorus*. Genome comparison of selected *Francisella* symbionts together with physiological experiments strongly suggest their important role in conferring advantages for the tick fitness, mainly providing B vitamins (Gerhart et al. 2016; Duron et al. 2018).

Multiple essential roles, including B vitamins provision, were also hypothesized for the symbiont ‘*Candidatus* Midichloria mitochondrii’ (hereafter *M. mitochondrii*) (Sassera et al. 2011; Olivieri et al. 2019). This bacterial endosymbiont was originally described in one of the most widespread ticks in Europe, *Ixodes ricinus*, and later reported in several other tick species from different continents (Beninati et al. 2004, 2009; Sacchi et al. 2004; Epis et al. 2008; Cafiso et al. 2016).

Interestingly, recent phylogenetic investigations revealed the occurrence of regular transitions between endosymbiotic and pathogenic forms during the course of evolution, such as *Coxiella burnetii* that seems to have recently evolved from a *Coxiella* endosymbiont ancestor (Duron et al. 2015) or conversely *Francisella* endosymbionts that probably originated from a pathogenic ancestor (Gerhart et al. 2016, 2018).

The well-known relevance of symbionts of arthropods on the host physiology and the nested interactions that can develop among symbionts and pathogens call for further investigation. For these reasons, the aims of this work were: (i) to update the knowledge on the prevalence, distribution and molecular characterization of selected TBPs and symbionts in different ecological zones in Africa, and (ii) to evaluate the patterns of co-infections detecting eventual competitive or facilitative interactions.

## Materials and methods

### Study sites, tick collection and identification

**Fig. 1:**
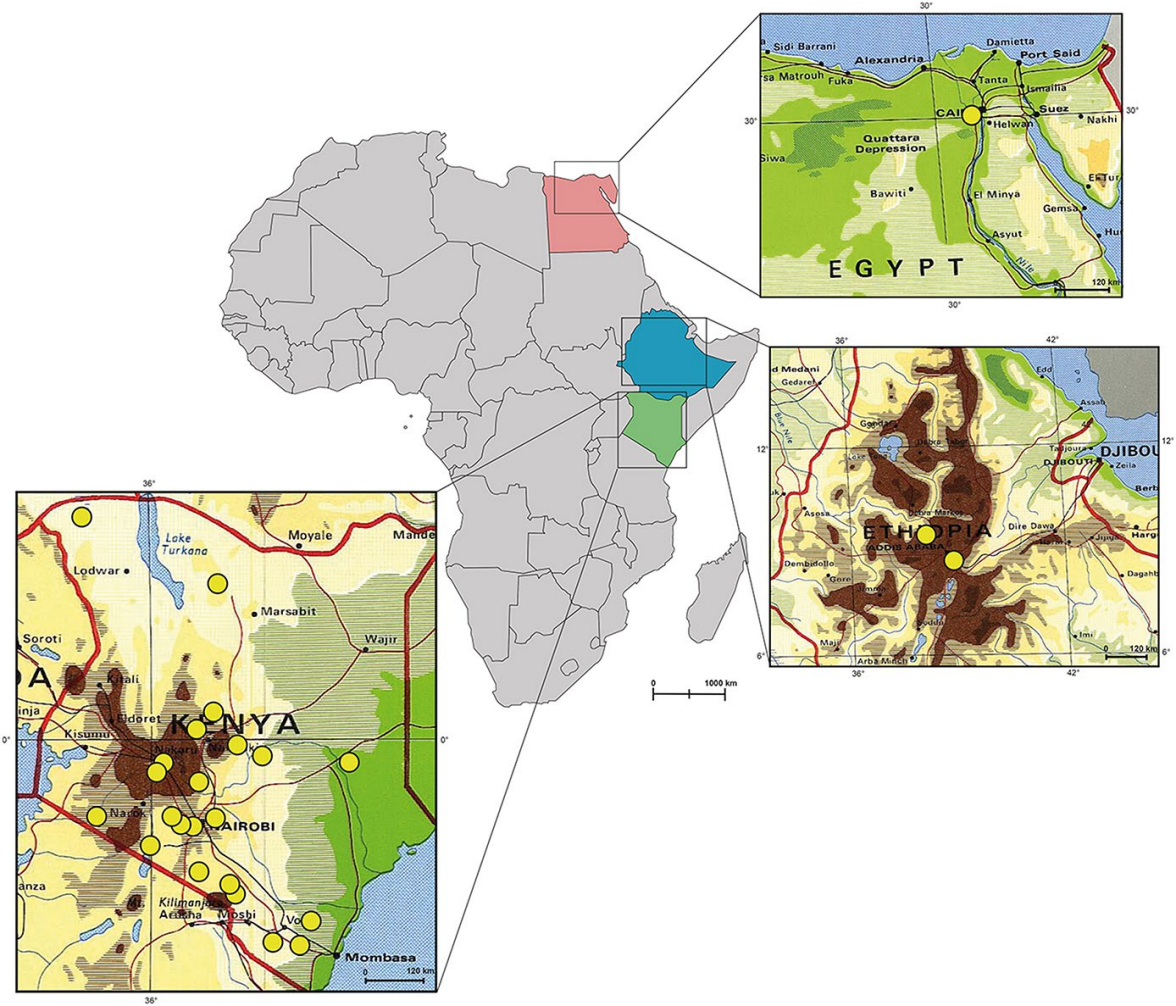
Political map of Africa indicating the countries where ticks were collected (i.e., Egypt in pink, Ethiopia in light-blue and Kenya in light-green). Insets show the localities where the ticks were collected. (Color figure online)

From 2009 to 2017 ticks were collected in various locations in Kenya from sympatric wild (African elephant, African buffalo, black and white rhinoceros, bongo antelope, dromedary camel, giraffe, hyena, lion, leopard, zebra and Grévy’s zebra) and domestic animals (cattle, sheep). Most of the samples were collected during routine veterinary surveillances of the Kenya Wildlife Service (KWS) performed in national parks, reserves, game reserves and from the vegetation (Fig. 1). Ticks were additionally collected in two districts in Ethiopia from cattle and sheep, where animals are managed under an extensive farming system at communal grazing land shared among small scale farmers. An additional portion of the dataset was collected from dogs living in close proximity with domestic ruminants in a single location in Egypt (Fig. 1). For each sampling point, the ecological zone values were extracted from the African ecological zones layer (AEZs; HarvestChoice 2011), by using the QGIS 3. According to this database, the samples were located in six agro-ecological zones. Collected ticks were preserved in vials containing 70% ethanol and morphologically identified using standard taxonomic keys (Theiler and Salisbury 1959; Walker et al. 2003).

### Molecular analyses

Genomic DNA was extracted individually from 339 ticks using the NucleoSpin® Tissue Kit (Macherey Nagel, Duren, Germany), according to the manufacturer’s instructions. The DNA quality was tested on a random subset of 68 samples (20%) using PCR amplification of tick mitochondrial ribosomal small RNA gene (12S rRNA) using a previously described protocol (Beati and Keirans 2001) (Additional file 1: Table S1).

The DNA samples were then tested by PCR for the presence of *Rickettsia* spp., *Anaplasma* spp./*Ehrlichia* spp., *Borrelia burgdorferi* (s.l.), *Babesia* spp./*Theileria* spp., *Coxiella* spp., *Midichloria* and *Francisella* using primers and conditions previously described (Additional file 1: Table S1). Positive PCR products of the expected size were extracted from agarose gel, purified using the QIAquick® Gel Extraction Kit (Qiagen, Hilden, Germany) following the manufacturer’s instructions. Purified DNA was sequenced with forward and reverse amplification primers (Eurofins Genomics, Ebersberg, Germany). Sequences were manually verified with Chromas Lite (Technelysium, Australia) and compared with those available in GenBank database using Basic Local Alignment Search Tool (BLAST: http://www.ncbi.nlm. nih.gov/BLAST). All the consensus sequences obtained in this study were deposited in GenBank database under the accession numbers given in the Additional file 2.

### Phylogenetic analyses

For the phylogenetic analyses on the sequences obtained in this study, two approaches were employed depending on the kind of sequence amplified. For each of the assays targeting SSU rRNA gene (*Anaplasma*/*Ehrlichia*, *Coxiella*, *Midichloria*, *Babesia*/*Theileria*), the newly obtained sequences were aligned on the SSU rRNA SILVA 128 Ref NR 99 database (Quast et al. 2012) with the ARB software package (Westram et al. 2011). After selection of similar sequences, the alignments were manually edited to optimize base-pairing in the predicted stems of the rRNA, and trimmed at both ends to the length of the amplicon sequences (i.e., excluding flanking regions present only in the database-derived sequences). For all other non-SSU assays (the three *Rickettsia* genes, *Borrelia* and *Francisella*), the sequences were directly aligned with selected database sequences using MUSCLE (Edgar 2004), and polished with Gblocks (Talavera and Castresana 2007).

For each final alignment thereby obtained, nucleotide substitution models were ranked according to the Akaike’s Information Criterion with jModeltest (Darriba et al. 2012). After model selection, maximum likelihood phylogenetic analyses were performed using phyML (Guindon and Gascuel 2003) with 100 bootstrap pseudo-replicates.

### Microorganisms co‐presence and ecological network inference

An *ad-hoc* script in R (R Core Team 2019) has been developed (available at https://github.com/MontagnaLab/co-presence_test) for testing whether the co-presence/co-absence of two microorganisms in the same tick individual is due to chance. This hypothesis has been tested simulating a null model (representing the hypothesis that co-presence of the same microorganism in individuals is due to chance) developed permuting the columns of a presence/absence matrix obtained for each couple of microorganisms based on PCR assays results (21 matrices in total). Each matrix was permuted 9999 times and the number of co-presences of each couple of microorganisms estimated for each permuted matrix. A two-tailed test with α/2 = 2.5% was performed for testing the null hypothesis. The values corresponding to the 2.5th and 97.5th percentiles of the simulated distribution were estimated. The number of co-presences observed for each couple of microorganisms in the total number of screened ticks was than calculated from the real presence/absence matrix. The null hypothesis is accepted when the observed value of co-presence was included between the values corresponding to the 2.5th and the 97.5th percentiles of the simulated distribution. In the event that the null hypothesis was rejected a p-value was calculated.

The relation between each tick-borne microorganism, tick species and vertebrate host was analysed and visualized by constructing a bipartite ecological network. Nodes of the network represent the vertebrate host and the tick species, whereas the edges represent the presence of individuals of the tick species on the vertebrate host. In addition, the information of the percentage of carried microorganisms was plotted as pie charts for each tick species. The network visualization was carried out using Cytoscape v.3.7.1 by importing the nodes and edges data mentioned above (Shannon et al. 2003).

## Results

In total, 339 ticks belonging to the Ixodidae family were collected. The ticks were morphologically identified as belonging to six genera: *Amblyomma, Dermacentor, Haemaphysalis, Hyalomma, Ixodes*, *Rhipicephalus*, for a total of 30 tick species. Additional information on tick species identification, number, gender, collection sites and hosts is listed in Table 1.

**Table 1:** Summary of tick species collected in this study, with details of geographical origin, sample size, gender, source of collection (host/vegetation) and agro-ecological zones (AEZ)

Tick species	Country	District	Site coordinates (latitude, longitude)	AEZ^a^ codes	Animal host/vegetation^b^	No. tick collected (m = male, f = female)
*Amblyomma cohaerens*	Kenya	Masai Mara	−1.404167, 34.941083	323	White Rhinoceros (*Ceratotherium simum*) Black Rhinoceros (*Diceros bicornis*)	10 (6m, 4f) 8 (4m,4f)
*Amblyomma eburneum*	Kenya	Meru National Park	0.036278, 38.220472	313	White Rhinoceros (*Ceratotherium simum*)	8 (4m,4f)
*Amblyomma gemma*	Kenya	Nairobi National Park	−1.368972, 36.795750	323	White Rhinoceros (*Ceratotherium simum*)	10 (3m,7f)
	Ethiopia	Boset	8.546778, 39.474833	322	Cow (*Bos taurus*)	11 (10m,1f)
*Amblyomma lepidum*	Kenya	Lotikipi	4.297389, 34.958611	312	Cow (*Bos taurus*)	7 (6m,1f)
*Amblyomma nuttalli*	Kenya	Masai Mara	−1.404167, 34.941083	323	White Rhinoceros (*Ceratotherium simum*)	4 (2m,2f)
*Amblyomma personatum*	Kenya	Lake Nakuru National Park	−0.316417, 36.119750	323	White Rhinoceros (*Ceratotherium simum*)	6 (6m)
*Amblyomma tholloni*	Kenya	Amboseli NP	−2.686077, 37.267332	313	Elephant (*Loxodonta africana*)	8 (5m,3f)
*Amblyomma variegatum*	Kenya	Lake Nakuru National Park	−0.316417, 36.119750	323	White Rhinoceros (*Ceratotherium simum*)	12 (11m,1f)
	Ethiopia	Ada’a	8.712361, 38.965556	323	Cow (*Bos taurus*)	17 (10m,7f)
*Dermacentor rhinocerinus*	Kenya	Chyulu	−2.551750, 37.797333	323	White Rhinoceros (*Ceratotherium simum*)	4 (1m,3f)
*Haemaphysalis* sp.	Kenya	Kimana	−2.739194, 37.525139	312	Lion (*Panthera leo*)	11 (8m,3f)
*Hyalomma albiparmatum*	Kenya	Masai Mara	−1.404167, 34.941083	323	White Rhinoceros (*Ceratotherium simum*)	6 (4m,2f)
	Kenya	Nairobi National Park	−1.368972, 36.795750	323	White Rhinoceros (*Ceratotherium simum*)	1 (1m)
	Kenya	Ngurumani	−1.958500, 36.072139	312	Zebra (*Equus quagga*)	3 (3m)
*Hyalomma dromedarii*	Kenya	Garissa	−0.506611, 39.671833	311	Camel (*Camelus dromedarius*)	4 (3m,1f)
*Hyalomma impeltatum*	Kenya	West Gate	0.753306, 37.344722	313	Grevy Zebra (*Equus grevyi*)	7 (3m,4f)
*Hyalomma rufipes*	Kenya	Lotikipi	4.297389, 34.958611	312	Cow (*Bos taurus*)	7 (6m,1f)
	Kenya	Kibiko	−1.291194, 36.674583	323	Giraffe (*Giraffa camelopardalis*)	3 (3m)
	Kenya	Tsavo East Kulalu	−3.068972, 39.326333	312	Buffalo (*Syncerus caffer*)	5 (4m,1f)
	Ethiopia	Ada’a	8.712361, 38.965556	323	Cow (*Bos taurus*)	15 (10m,5f)
*Hyalomma truncatum*	Kenya	West Gate	0.753306, 37.344722	313	Grevy Zebra (*Equus grevyi*)	5 (4m,1f)
	Ethiopia	Ada’a	8.712361, 38.965556	323	Cow (*Bos taurus*)	19 (10m,9f)
*Ixodes* sp.	Kenya	Mt Kenya conservancy	−0.180833, 37.550778	323	Bongo (*Tragelaphus eurycerus*)	1 (1f)
*Rhipicephalus appendiculatus*	Kenya	Mt Kenya conservancy	−0.180833, 37.550778	323	Bongo (*Tragelaphus eurycerus*)	9 (5m,4f)
*Rhipicephalus camicasi*	Egypt	Giza Cairo	29.970639, 31.141056	211	Dog (*Canis lupus familiaris*)	2 (2m)
*Rhipicephalus carnivoralis*	Kenya	Ewaso Kendong	−1.155694, 36.506083	323	Leopard (*Panthera pardus*)	4 (3m,1f)
*Rhipicephalus compositus*	Kenya	Nairobi National Park	−1.368972, 36.795750	323	White Rhinoceros (*Ceratotherium simum*)	7 (7m)
*Rhipicephalus decoloratus*	Ethiopia	Ada’a	8.712361, 38.965556	323	Cow (*Bos taurus*)	12 (2m,10f)
*Rhipicephalus evertsievertsi*	Kenya	Lotikipi	4.297389, 34.958611	312	Cow (*Bos taurus*)	4 (4f)
	Ethiopia	Boset	8.546778, 39.474833	322	Cow (*Bos taurus*)	7 (5m,2f)
*Rhipicephalus humeralis*	Kenya	Sagala	−3.530833, 38.669972	312	Buffalo (*Syncerus caffer*)	4 (4m)
*Rhipicephalus maculatus*	Kenya	Mbirikani	−2.756000, 37.774667	313	Vegetation (Savannah grassland)	8 (5m,3f)
*Rhipicephalus muelensi*	Kenya	Bachuma	−3.576361, 38.938000	312	Cow (*Bos taurus*)	4 (1m,3f)
*Rhipicephalus praetextatus*	Kenya	Ol jogi	0.312389, 36.976083	323	White Rhinoceros (*Ceratotherium simum*)	8 (5m,3f)
	Kenya	Machakos	−1.524611, 37.246361	323	Hyena (*Crocuta crocuta*)	4 (3m,1f)
	Ethiopia	Ada’a	8.712361, 38.965556	323	Cow (*Bos taurus*)	20 (10m,10f)
*Rhipicephalus pravus*	Kenya	Lotikipi	4.297389, 34.958611	312	Sheep (*Ovis aries*)	6 (4m,2f)
*Rhipicephalus pulchellus*	Kenya	Nairobi National Park	−1.368972, 36.795750	323	Black rhino (*Diceros bicornis*)	8 (4m,4f)
	Ethiopia	Boset	8.546778, 39.474833	323	Cow (*Bos taurus*)	20 (10m,10f)
*Rhipicephalus sanguineus* s.l.	Egypt	Giza Cairo	29.970639, 31.141056	211	Dog (*Canis lupus familiaris*)	18 (1m,17f)
*Rhipicephalus* sp.	Kenya	Murararandia	−0.730306, 36.909667	323	Cow (*Bos taurus*)	2 (2m)

^a^AEZ code refers to agro-ecological zone according to https://harvestchoice.org/maps/agro-ecological-zones-sub-saharan-africa as follows: 211: subtropical–warm, arid; 311: tropical–warm, arid; 312: tropical–warm, semiarid; 313: tropical–warm, subhumid; 322: tropical–cool, semiarid; 323: tropical–cool, subhumid

^b^All the adult ticks collected from the host (n = 331) were at different stages of engorgement, whereas those collected from vegetation (n = 8) were unfed

Molecular screening revealed the presence of pathogens belonging to the genera *Rickettsia*, *Anaplasma*, *Ehrlichia*, *Borrelia*, *Babesia*, and *Theileria*, with *Rickettsia* bacteria being the most widespread. Indeed, *Rickettsia* spp. were found in 18 out of 339 ticks tested (5.3%). Subsequent analyses of the *gltA* gene sequences revealed that 10 out of 18 rickettsias share high identity with *Rickettsia aeschlimannii* (detected in 9/30 *Hy. rufipes* and 1/7 *Hy. impeltatum*). The *gltA* marker did not allow to discriminate the remaining eight *Rickettsia* sequences at the species level (Additional file 2: Fig. S1a). Thus, additional sequencing of *ompA* and *ompB* genes of a representative subset of positive samples was performed, and, besides confirming that the most prevalent species was *R. aeschlimannii* (n = 10), allowed to identify other rickettsial species: *R. africae* (n = 5) detected in *Am. gemma* (n = 2), *Am. variegatum* (n = 2) and *Hy. impeltatum* (n = 1); *R. massiliae* (n = 2) in *Rh. praetextatus*; and one *R. rhipicephali* in *Am. cohaerens* (Additional file 2: Fig. S1b,c).

*Anaplasma* spp. DNA was detected in 2.1% (7/339) of the ticks. The phylogenetic analysis based on 16S rRNA sequences did not provide sufficient discriminatory power to clarify the species assignment. However, the obtained sequences formed two distinct clusters: the sequences from three *Rh. pravus* and two *Rh. decoloratus* ticks clustered with *A. marginale, A. centrale* and *A. ovis* sequences downloaded from NCBI with 100% bootstrap support; whereas two sequences, from *Am. variegatum* and *Rh. decoloratus*, clustered with *A. platys* (74% bootstrap support) (Additional file 2: Fig. S2).

*Ehrlichia* bacteria were detected in three ticks only (0.9% of the total, one *Am. variegatum*, one *Am. lepidum*, one *Hy. impeltatum*), collected from cattle and Grevy’s zebra. The sequences showed 100% of identity with *E. ruminantium* (GenBank: NR074155), supported with 100% bootstrap in the phylogeny (Additional file 2: Fig. S2).

Different *Theileria* spp. were detected in seven out of 339 ticks (2.1%). Among these, three were clearly identified as *T. taurotragi* (detected in two *Rh. appendiculatus* collected from antelope) and as *T. velifera* (detected in an *Am. cohaerens* collected from Black rhinoceros). Two sequences, from *Am. cohaerens* and *Am. gemma* collected from white rhinoceros, clustered together with an unknown *Theileria* species detected in cheetahs in the same area in 2009 (Githaka et al. 2012). One *Theileria* sequence detected in *Rh. pulchellus* collected from black rhinoceros clustered together with another unknown *Theileria* sp. detected in blood samples from giraffes in the same area in 2011 (GenBank AB650504). Finally, a *Theileria* sequence detected in *Am. cohaerens* collected from white rhinoceros likely represents a new species, showing only 89.97% nucleotide identity with *T. mutans* (GenBank: JN572694). However, additional characterization would be required as it is not possible to establish new variants of piroplasms based only on the use the 18S rRNA gene (Chae et al. 1999; Allsopp and Allsopp 2006) (see Additional file 2: Fig. S3 for the phylogeny of the *Theileria*).

*Babesia* was detected in two ticks (0.6%). The sequence obtained from *Am. variegatum* shows 98% of identity with *B. caballi* (GenBank: MH424325) and the one from *Hy. rufipes* shows 100% identity with *B. occultans* (GenBank: MH899757). Both identifications were highly supported in the phylogeny (Additional file 2: Fig. S3).

*Borrelia* positivity was detected in only one tick (*Hy. rufipes*). The obtained ITS sequence shows 100% identity with *B. garinii* from an *Ixodes ricinus* sample in Finland (GenBank: MG356954). Consistently, the novel sequence results embedded in a clade of *B. garinii* in a phylogenetic analysis (Additional file 2: Fig. S4).

Molecular screening of bacterial symbionts revealed the presence of *Coxiella*, *Francisella* and *Midichloria* across the tick populations. The most prevalent endosymbionts were *Coxiella* spp., successfully amplified from 95 of the 339 ticks tested (28%). Putative *Coxiella* endosymbionts were found among 16 tick species, whereas only one *Coxiella* strain identical to the pathogenic *Coxiella burnetii* was detected in one specimen of *Rh. pulchellus*. (Table 2). Phylogenetic analysis based on the 16S rRNA gene showed, in most cases, that closely related *Coxiella* strains are found in closely related tick species (Additional file 2: Fig. S5).

**Table 2:** Prevalence (%) of endosymbionts found in collected ticks. In parentheses: no. postive/no. examined

Tick species	*Coxiella* sp.			*Francisella* sp.			*Midichloria* sp.			*Rickettsia* sp.		
	Female	Male	Total	Female	Male	Total	Female	Male	Total	Female	Male	Total
*Amblyomma cohaerens*	100 (8/8)	80 (8/10)	88.9 (16/18)	− (0/8)	− (0/10)	− (0/18)	12.5 (1/8)	− (0/10)	5.6 (1/18)	− (0/8)	10 (1/10)	5.6 (1/18)
*Amblyomma eburneum*	− (0/4)	− (0/4)	− (0/8)	− (0/4)	− (0/4)	− (0/8)	− (0/4)	25 (1/4)	12.5 (1/8)	− (0/4)	− (0/4)	− (0/8)
*Amblyomma gemma*	25 (1/4)	(0/17)	4.8 (1/21)	− (0/4)	− (0/17)	− (0/21)	− (0/4)	(0/17)	− (0/21)	− (0/4)	11.8 (2/17)	9.5 (2/21)
*Amblyomma lepidum*	100 (1/1)	− (0/6)	− (0/7)	− (0/1)	− (0/6)	− (0/7)	− (0/1)	− (0/6)	14.3 (1/7)	− (0/1)	− (0/6)	− (0/7)
*Amblyomma nuttalli*	− (0/2)	− (0/2)	− (0/4)	− (0/2)	− (0/2)	− (0/4)	− (0/2)	− (0/2)	− (0/4)	− (0/2)	− (0/2)	− (0/4)
*Amblyomma personatum*	–	100 (6/6)	100 (6/6)	–	− (0/6)	− (0/6)	–	16.7 (1/6)	16.7 (1/6)	–	− (0/6)	− (0/6)
*Amblyomma tholloni*	100 (5/5)	100 (3/3)	100 (8/8)	− (0/5)	− (0/3)	− (0/8)	− (0/5)	− (0/3)	− (0/8)	− (0/5)	− (0/3)	− (0/8)
*Amblyomma variegatum*	− 0/8	38 (8/21)	27.6 (8/29)	− 0/8	− (0/21)	− (0/29)	− 0/8	− (1/21)	3.4 (1/29)	− 0/8	− (0/21)	6.9 (2/29)
*Dermacentor rhinocerinus*	− (0/3)	− (0/1)	− (0/4)	66.7 (2/3)	− (0/1)	50 (2/4)	− (0/3)	− (0/1)	− (0/4)	− (0/3)	− (0/1)	− (0/4)
*Haemaphysalis* sp.	33.3 (1/3)	25 (2/8)	27.3 (3/11)	− (0/3)	− (0/8)	− (0/11)	− (0/3)	− (0/8)	− (0/11)	− (0/3)	− (0/8)	− (0/11)
*Hyalomma albiparmatum*	− (0/2)	− (0/8)	− (0/10)	− (0/2)	25 (2/8)	20 (2/10)	− (0/2)	− (0/8)	− (0/10)	− (0/2)	− (0/8)	− (0/10)
*Hyalomma dromedarii*	− (0/1)	− (0/3)	− (0/4)	− (0/1)	− (0/3)	− (0/4)	− (0/1)	− (0/3)	− (0/4)	− (0/1)	− (0/3)	− (0/4)
*Hyalomma impeltatum*	− (0/4)	− (0/3)	− (0/7)	50 (2/4)	− (0/3)	28.6 (2/7)	− (0/4)	− (0/3)	− (0/7)	25 (1/4)	33.3 (1/3)	28.6 (2/7)
*Hyalomma rufipes*	− (0/7)	− (0/23)	− (0/30)	57 (4/7)	47.8 (11/23)	50 (15/30)	42.8 (3/7)	21.7 (5/23)	26.7 (8/30)	28.6 (2/7)	30.4 (7/23)	30 (9/30)
*Hyalomma truncatum*	− (0/10)	7.1 (1/14)	4.2 (1/24)	50 (5/10)	28.6 (4/14)	37.5 (9/24)	− (0/10)	− (0/14)	− (0/24)	− (0/10)	− (0/14)	− (0/24)
*Ixodes* sp.	–	− (0/1)	− (0/1)	–	− (0/1)	− (0/1)	–	− (0/1)	− (0/1)	–	− (0/1)	− (0/1)
*Rhipicephalus appendiculatus*	100 (4/4)	80 (4/5)	88.9 (8/9)	− (0/4)	− (0/5)	− (0/9)	− (0/4)	− (0/5)	− (0/9)	− (0/4)	− (0/5)	− (0/9)
*Rhipicephalus camicasi*	–	− (0/2)	− (0/2)	–	− (0/2)	− (0/2)	–	− (0/2)	− (0/2)	–	− (0/2)	− (0/2)
*Rhipicephalus carnivoralis*	100 (1/1)	66.7 (2/3)	75 (3/4)	− (0/1)	− (0/3)	− (0/4)	− (0/1)	− (0/3)	− (0/4)	− (0/1)	− (0/3)	− (0/4)
*Rhipicephalus compositus*	–	100 (7/7)	100 (7/7)	–	− (0/7)	− (0/7)	–	− (0/7)	− (0/7)	–	− (0/7)	− (0/7)
*Rhipicephalus decoloratus*	− (0/10)	− (0/2)	− (0/12)	− (0/10)	− (0/2)	− (0/12)	− (0/10)	− (0/2)	− (0/12)	− (0/10)	− (0/2)	− (0/12)
*Rhipicephalus evertsievertsi*	83.3 (5/6)	80 (4/5)	81.9 (9/11)	− (0/6)	20 (1/5)	9.1 (1/11)	− (0/6)	− (0/5)	− (0/11)	− (0/6)	− (0/5)	− (0/11)
*Rhipicephalus humeralis*	–	− (0/4)	− (0/4)	–	− (0/4)	− (0/4)	–	− (0/4)	− (0/4)	–	− (0/4)	− (0/4)
*Rhipicephalus maculatus*	66.7 (2/3)	60 (3/5)	62.5 (5/8)	− (0/3)	− (0/5)	− (0/8)	− (0/3)	− (0/5)	− (0/8)	− (0/3)	− (0/5)	− (0/8)
*Rhipicephalus muelensi*	− (0/3)	− (0/1)	− (0/4)	− (0/3)	− (0/1)	− (0/4)	− (0/3)	− (0/1)	− (0/4)	− (0/3)	− (0/1)	− (0/4)
*Rhipicephalus praetextatus*	42.8 (6/14)	33.3 (6/18)	37.5 (12/32)	7.1 (1/14)	− (0/18)	16.7 (1/6)	7.1 (1/14)	22.2 (4/18)	15.6 (5/32)	7.1 (1/14)	5.5 (1/18)	6.2 (2/32)
*Rhipicephalus pravus*	− (0/2)	100 (4/4)	66.7 (4/6)	− (0/2)	− (0/4)	− (0/6)	− (0/2)	− (0/4)	− (0/6)	− (0/2)	− (0/4)	− (0/6)
*Rhipicephalus pulchellus*	− (0/14)	− (0/14)	− (0/28)	− (0/14)	− (0/14)	− (0/28)	− (0/14)	− (0/14)	− (0/28)	− (0/14)	− (0/14)	− (0/28)
*Rhipicephalus sanguineus* s.l.	11.8 (2/17)	− (0/1)	11.1 (2/18)	− (0/17)	− (0/1)	− (0/18)	35.3 (6/17)	− (0/1)	33.3 (6/18)	− (0/17)	− (0/1)	− (0/18)
*Rhipicephalus* sp.	–	50 (1/2)	50 (1/2)	–	− (0/2)	− (0/2)	–	− (0/2)	− (0/2)	–	− (0/2)	− (0/2)

*Francisella* spp. were detected in 32 ticks out of 339 tested (9.4%). *Francisella* positive ticks belonged to seven tick species, mainly within the *Hyalomma* genus, in which the prevalence was high, ranging from 20 to 50% (Table 2). Although the phylogenetic analysis of the *rpo*B gene was poorly informative in terms of species determination, it still allowed to identify the detected organisms as members of the *Francisella-*like endosymbionts (FLE) clade, and genetically distant from strains of pathogenic *Francisella* species and subspecies. In addition, all of the sequences of FLE detected in *Hyalomma* ticks were closely related, whereas FLE detected in *De. rhinocerinus* and *Rh. praetextatus* clustered together with a distinct, long branch, probably due to higher sequence divergence (Additional file 2: Fig. S6).

A total of 24 ticks out of 339 (7.1%) were positive for *Midichloria*. The rate of infection among specimens of the positive tick species was generally lower compared to *Coxiella* and *Francisella* endosymbionts. However, *Midichloria* resulted the most prevalent symbiont of *Rh. sanguineus* s.l., reaching an infection rate of 33.3% versus 11.1% of *Coxiella* endosymbionts, and the two symbionts were never detected in the same individual (Table 2). On the other hand, the phylogenetic tree clearly showed that similar sequences of *Midichloria* are found in genetically distant tick species, with the most diverging *Midichloria* member identified in *Am. lepidum* (Additional file 2: Fig. S7).

**Fig. 2:**
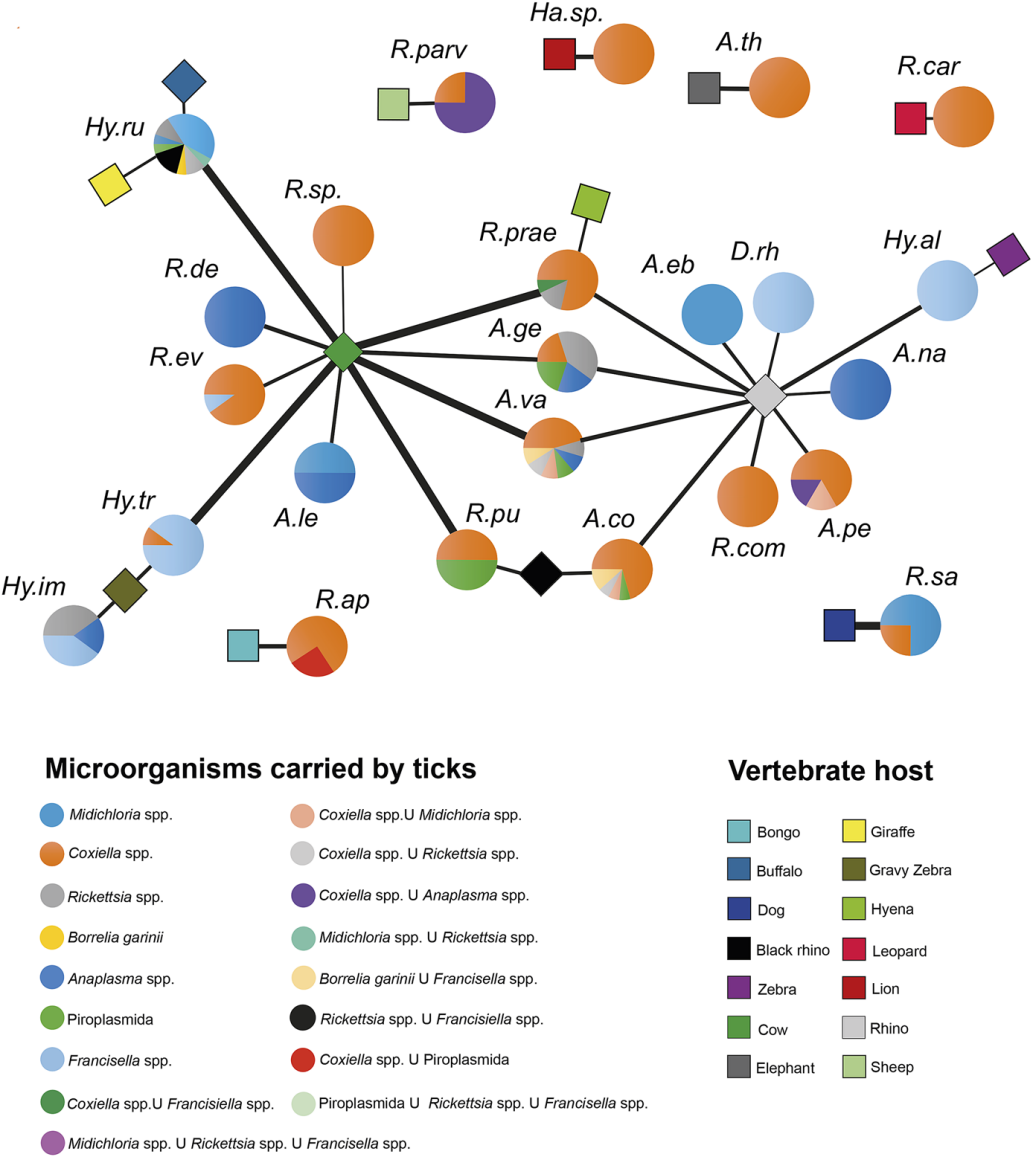
Bipartite ecological network showing the relation among tick-borne microorganism, tick species and its vertebrate host. The vertebrate host and the tick species, the nodes of the network, are represented in the form of squares and circles, respectively; whereas edges represent the associations between the tick species and their vertebrate host. For each tick species, the relative abundance (expressed as percentage) of microorganisms detected through the PCR screening of individuals is reported in pie charts. A.co, *Amblyomma cohaerens*; A.eb, *Amblyomma eburneum*; A.ge, *Amblyomma gemma*; A.le, *Amblyomma lepidum*; A.nu, *Amblyomma nuttalli*; A.pe, *Amblyomma personatum*; A.th, *Amblyomma tholloni*; A.va, *Amblyomma variegatum*; D.rh, *Dermacentor rhinocerinus*; Ha.sp, *Haemaphysalis* sp; Hy.al, *Hyalomma albiparmatum*; Hy.dr, *Hyalomma dromedarii*; Hy.im, *Hyalomma impeltatum*; Hy.ru, *Hyalomma rufipes*; Hy.tr, *Hyalomma truncatum*; I.sp, *Ixodes* sp.; R.ap, *Rhipicephalus appendiculatus*; R.cam, *Rhipicephalus camicasi*; R.car, *Rhipicephalus carnivoralis*; R.com, *Rhipicephalus compositus*; R.de, *Rhipicephalus decoloratus*; R.ev, *Rhipicephalus evertsievertsi*; R.hu, *Rhipicephalus humeralis*; R.mu, *Rhipicephalus muelensi*; R.prae, *Rhipicephalus praetextatus*; R.prav, *Rhipicephalus pravus*; R.pu, *Rhipicephalus pulchellus*; R.sa, *Rhipicephalus sanguineus*; R.sp., *Rhipicephalus* sp

Interestingly, co-infections were spotted: 21 ticks resulted infected with more than one microorganism, including 16 double infections with seven combinations and five triple infections, mainly involving *Midichloria*, *Francisella* and *Rickettsia* (Table 3). Co-infection between tick-borne microorganisms occurred more frequently in generalist tick species with a broad host spectrum, such a*s Hy. rufipe*s and* Am. variegatum*, whereas ticks with a pronounced host specificity, such as* Am. tholloni* and *Rh. carnivoralis*, resulted mainly bearing single microorganisms, especially vertically transmitted endosymbionts (Fig. 2).

**Table 3:** Tick-borne pathogens and endosymbionts co-infections in ticks tested (n = 339)

Tick-borne microorganism	Positive		Tick species (no. positive specimens; m = male, f = female ), host
	No.	Prevalence (%)	
Dual infection	16	4.7	
*Coxiella* + *Anaplasma*	4	1.2	*Rh. pravus* (3m), sheep *Am. variegatum* (1m), white rhinoceros
*Coxiella* + *Theileria*	4	1.2	*Am. cohaerens* (2f), white rhinoceros *Rh. appendiculatus* (2f)
*Coxiella* + *Midichloria*	4	1.2	*Am. cohaerens* (1f), white rhinoceros *Am. personatum* (1m), white rhinoceros *Am. variegatum* (1m), white rhinoceros *Rh. praetextatus* (1m), cow
*Coxiella* + *Rickettsia*	1	0.3	*Am. variegatum* (1m), white rhinoceros
*Coxiella* + *Francisella*	1	0.3	*Rh. praetextatus* (1f), white rhinoceros
*Midichloria* + *Rickettsia*	1	0.3	*Hy. rufipes* (1m), cow
*Midichloria* + *Francisella*	1	0.3	*Hy. rufipes* (1f), cow
Triple infection	5	1.5	
*Midichloria* + *Francisella* + *Rickettsia*	4	1.2	*Hy. rufipes* (2f, 2 m), cow
*Babesia* + *Francisella* + *Rickettsia*	1	0.3	*Hy. rufipes* (1m), cow
Total	21	6.2	

Furthermore, comparing the null model distribution with the observed values of co-presence, the association between *Rickettsia* and *Francisella* in the same host tick resulted positively significant (p < 0.01) (Additional file 3: Fig. S8 A), this association was often observed in *Hy. rufipes* individuals (Fig. 2). Through the same analysis, *Francisella* and *Coxiella* association was found to be negatively significant (p < 0.001) (Additional file 3: Fig. S8 B).

## Discussion

Spotted fever group (SFG) rickettsioses are the most frequently tick-borne diseases recognised among travellers returning from sub-Saharan Africa with acute febrile illness, this is indicative of the endemicity of rickettsial diseases in African countries and their impact on public health (Freedman et al. 2006; Parola et al. 2013). *Rickettsia africae*, *R. aeschlimannii* and *R. massiliae*, all detected in this investigation, are considered among the main pathogenic SFG rickettsiae (Parola 2006). Our results are in agreement with those of previous studies, which identified several tick species as potential vectors for these rickettsiae in multiple sub-Saharan African countries. Indeed *Hyalomma* ticks frequently harbour *R. aeschlimanni*, especially *Hy. rufipes* and *Hy. marginatum* (Mura et al. 2008; Kumsa et al. 2015; Azagi et al. 2017). On the other side, our finding of *R*. *africae* in multiple *Amblyomma* species, with higher prevalence in *Am. variegatum* and *Am. gemma*, confirms previous findings (Jensenius et al. 2003; Macaluso et al. 2003; Mediannikov et al. 2010; Mutai et al. 2013; Vanegas et al. 2018). The geographical distribution of these SFG rickettsiae strongly overlaps with the distribution of their respective tick vectors.

In the last years several SFG rickettsial species that are pathogenic for the vertebrate hosts have also been identified as secondary tick symbionts, reaching a high frequency of infection in some tick populations, enhancing the host fitness and being transovarially transmitted to the offspring, e.g., *Rickettsia parkeri* or *R. monacensis* (Ahantarig et al. 2013). Whether the three rickettsial species detected here play a similar role in their host remains an open question.

A noteworthy finding for human health is the unusual detection of *B. garinii* DNA in a *Hy. rufipes* tick collected from a Giraffe in Kenya. *Borrelia garinii* is one of the predominant genospecies of the *B. burgdorferi* sensu lato complex, known to cause Lyme disease in Europe, and is considered the most neurotropic *Borrelia* spirochete (Benredjem et al. 2014; Stanek and Strle 2018). *Borrelia garinii* is usually vectored by *Ixodes* ticks in Europe and Asia, but was also reported in North Africa (Tunisia and Morocco) in association with *Ixodes* species (Bouattour et al. 2004), identified as *I. ricinus* by Bouattour, but possibly belonging to the subsequently described species *I. inopinatus* (Estrada-Pena et al. 2014). Birds are considered the main reservoirs and biological carriers of *B. garinii* (Comstedt et al. 2011; Pajoro et al. 2018). The role of migratory birds in the spread of this spirochete can explain the novel finding of the positivity of *Hy. rufipes*, a tick species that has been reported infesting various migratory birds worldwide (England et al. 2016). Based on this evidence, and on previous reports of *Borrelia lusitaniae* in *Hy. marginatum* (Michelis et al. 2000), these findings represent uncommon cases of *B. burgdorferi* sensu lato species associated with metastriate ticks (Margos et al. 2020). Considering that ticks can be infected following an infected blood meal, only further studies can confirm the vectorial competence of *Hyalomma* ticks for *Borrelia* species focusing on the acquisition, maintenance, and subsequent transmission into a vertebrate host during blood feeding.

Additionally, a high diversity of tick-borne pathogens relevant for domestic and wild animal health were here detected in the tick populations tested, although with low prevalence. Among others, we detected *E. ruminantium*, a bacterium mainly transmitted by ticks of the genus *Amblyomma*, causing heartwater disease affecting wild and domestic ruminants (Uilenberg 1997; Allsopp 2010). The occurrence of several piroplasm species, such as *B. caballi*, *B. occultans*, *T. taurotragi* and *T. velifera*, considered mildly to severely pathogenic with significant impact on animal health, is here reported, in accordance with previous surveys (de la Fuente et al. 2008; Sivakumar et al. 2014; Omondi et al. 2017).

The most retrieved symbiont was *Coxiella*, found in representatives of four out of six tick genera analysed, reaching high prevalence in many of the analysed species, especially within the *Rhipicephalus* and *Amblyomma* genera (Table 2). According to phylogenetic analysis based on the 16S rRNA gene sequence, most of the novel sequences result closely related to other *Coxiella* associated to tick species of the same genus. Moreover, although not fully supported, the deeper tree topology is overall consistent with the four *Coxiella* clades identified by Duron and colleagues through multilocus sequence typing (MLST) (Duron et al. 2015). Accordingly, whereas a great diversity exists within the genus, our results confirm the overall co-cladogenesis of *Coxiella* symbionts with their hosts, but, at the same time, presence of highly related *Coxiella* in unrelated ticks suggest relatively frequent host species shifts (Duron et al. 2015). These features likely reflect a long mutualistic coevolution, conferring significant advantages to both organisms, and with a certain degree of flexibility with respect to host/symbiont species.

The second most widespread symbiont is *Francisella.* Consistently with previous studies (Ivanov et al. 2011; Szigeti et al. 2014; Azagi et al. 2017; Duron et al. 2017), *Francisella* resulted highly prevalent among the *Hyalomma* species tested, but we additionally detected this bacterium in species in which it was never reported before (*Hy. impeltatum* and *Hy. albiparmatum*). The nutritional mutualism of *Francisella* can explain the negative correlation we found with *Coxiella* endosymbionts, since they provide the same benefit for the host (Duron et al. 2017, 2018). Indeed, in recent studies *Francisella* was defined as an alternative obligate symbiont to *Coxiella*, which appeared to be replaced by *Francisella* in multiple tick species (Duron et al. 2017). In our dataset *Francisella* was found to significantly co-occur with *Rickettsia*, as frequently reported previously across tick taxa (Scoles 2004; Ahantarig et al. 2013; Budachetri et al. 2015; Azagi et al. 2017), whereas *Coxiella* endosymbionts were often reported as single infections. Taken together, these data allow to hypothesize that *Francisella* is less competitive than the *Coxiella* primary symbiont, or that multiple co-occurring symbionts can act in conjunction or even synergistically.

Noteworthy, *Midichloria* is the most prevalent (33%) symbiont in *Rh. sanguineus* s.l. with *Coxiella* as second (11%). This finding is interesting when compared with a recent study on the microbial communities of various *Rh. sanguineus* s.l. populations in France, Arizona (USA) and Senegal, which indicated *Coxiella* and *Rickettsia* as the predominant endosymbionts, with strong geographical clustering (René-Martellet et al. 2017). In particular, René-Martellet and colleagues concluded that the relative abundance of these endosymbionts varies depending on the geographical origin and the lineage of the tick, with *Coxiella* strongly associated with Senegal ticks. We can add to the complex landscape of the symbionts of *Rh. sanguineus* s.l. the notion that in Egypt the predominant symbiont is neither *Coxiella*, nor *Rickettsia*, but *Midichloria*. These results confirm the lability of the bacterial community structure hosted by this tick species, much differently that what seen in most other species (Duron et al. 2017). There are various possible explanations, such as the influence of multiple ecological and geographical factors (Lalzar et al. 2014; Abraham et al. 2017; Bonnet et al. 2017) as well as the host-feeding behavior of the ticks, the host’s immune system and the direct interaction of protozoan or bacterial pathogens (Adegoke et al. 2020; Aivelo et al. 2019; Hawley and Altizer 2011). Alternatively, or in conjunction, the possibility that the analysed individuals belong to different sibling species of the *Rh. sanguineus* s.l. group must be considered (Dantas-Torres and Otranto 2015; Coimbra-Dores et al. 2020).

Despite the low prevalence of *Midichloria* symbionts in African ticks, the detection of similar sequences of *Midichloria* in genetically distant tick species provides additional support to the hypothesis of frequent horizontal transfers of these bacteria (Skarphédinsson et al. 2005; Bazzocchi et al. 2013; Cafiso et al. 2018; Di Lecce et al. 2018; Serra et al. 2018). Low genetic variation of *Midichloria* was commonly reported in surveys based on phylogenetic analysis of 16S rRNA gene sequences (Cafiso et al. 2016; Duron et al. 2017), whereas recent MLST-based studies provide evidence of co-evolution of *Midichloria* in some tick populations (Buysse and Duron 2018; Al-khafaji et al. 2019).

Additionally, we report a frequent albeit not statistically significant co-occurrence of *Midichloria* with *Rickettsia* in specimens of *Hy. rufipes.* We can draw a parallel with what recently reported in the tick *A. maculatum*, in which *R. parkeri* infection was found to promote *Midichloria* colonization in the midgut, salivary glands, and ovarian tissues of fed and unfed ticks, indicating a synergistic relationship between them (Budachetri et al. 2018).

## Conclusions

This study brings further attention to the complexity of ticks’ microbial communities and calls for an in-depth analysis of the interactions among the tick-borne microorganisms. Further multidisciplinary investigations involving metagenomics, genomics, and ecology are pivotal to better understand these dynamics, with possible important consequences on human and animal health, economy, and on the preservation of endangered species.

## Supplementary Information

Below is the link to the electronic supplementary material.

Electronic supplementary material 1 (DOC 74 kb)

Electronic supplementary material 2 (PDF 466 kb)

Electronic supplementary material 3 (DOCX 170 kb)
