# Supplementary material for: Multi‐country investigation of the diversity and associated microorganisms isolated from tick species from domestic animals, wildlife and vegetation in selected african countries
Source: Exp Appl Acarol. 2021 Mar 1;83(3):427–48. doi: 10.1007/s10493-021-00598-3 (PMC7940270; doi:10.1007/s10493-021-00598-3)
Supplement: Supplementary file 1 — Electronic supplementary material 1 (DOC 74 kb) [file 10493_2021_598_MOESM1_ESM.doc]

**Additional file 1: Table S1**. Target genes, PCR primers, amplicon sizes (bp) and references used in this study.

Briefly, the presence of *Rickettsia* spp. was determined by the amplification of the citrate synthase (*gltA*) gene (Roux et al., 1997). Additional PCR amplification of the outer membrane protein A gene (*ompA*) and outer membrane protein B gene (*ompB*) were performed on a representative subset of the positive samples to differentiate closely related *Rickettsia* species (Kimita et al., 2016). A semi-nested PCR was conducted for the simultaneously detection of *Anaplasma* and *Ehrlichia* 16S rRNA gene (Teshale et al., 2015). For the detection of *B. brugdorferi* (s.l.) was performed a nested PCR specific for the 5S–23S rRNA intergenic spacer gene, following the protocol of Chu et al. (2008). One set of primers was used to amplify a 408 bp fragment of the 18S rRNA gene of *Babesia spp.* and *Thelieria spp.*(Olmeda et al., 1997)*,* and the 16S rRNA gene of *Coxiella* spp. (Lalzar et al., 2012). The screening of *Midichloria* was performed using a semi-nested PCR following the protocol of Cafiso et al. (2016). For the detection of *Francisella* was performed a semi-nested PCR targeting the *rpoB* gene (Duron et al., 2017).

| **Target organism** | **Target gene** | | **Primer name** | **Primer sequence (5’-3’)^1^** | **Amplicon size (bp)** | **Reference** |
| --- | --- | --- | --- | --- | --- | --- |
| Tick species | *mitochondrial 12S* rRNA^a^ | T1B | | AAACTAGGATTAGATACCCT | 338 | Beati and Keirans, 2001 |
|  |  | T1A | | AATGAGAGCGACGGGCGATGT |  |  |
| *Rickettsia* | *GltA*^a^ | RpCS.877p | | GGGGACCTGCTCACGGCGG  ATTGCAAAAAGTACAGTGAACA | 1234 | Roux et al., 1997 |
|  |  | RpCS.1258n | |  |  |  |
|  | *Omp*B^b^ | RAK1009Fw | | ACATKGTTATACARAGTGYTAATGC | 444 | Kimita et al., 2016 |
|  |  | OmpB1902Rv | | CCGTCATTTCCAATAACTAACTC |  |  |
|  |  | RAK1452Rv | | ASGTTAACTTKACCGYTTATAACTGT |  |  |
|  | *Omp*A^b^ | OmpAM50Fw | | TTGCGTTATAACACTTTTTAAGTGA | 530 | Kimita et al., 2016 |
|  |  | OmpA642Rv | | ATTACCTATTGTTCCGTTAATGGCA: |  |  |
|  |  | 190-70Fw | | ATGGCGAATATTTCTCCAAAA |  |  |
|  |  | 190-701Rv | | GTTCCGTTAATGGCAGCATCT |  |  |
| *Anaplasmataceae* | 16S rRNA^a^ | EHR 16SD | | GGTACCYACAGAAGAAGTCC | 925 | Hornok et al., 2008  Teshale et al., 2018 |
|  |  | EBR2 | | TGCTGACTTGACATCATCCC |  |  |
|  |  | EBR3 | | TTGTAGTCGCCATTGTAGCAC |  |  |
| *Borrelia* *burgdorferi* sensu lato | 5S-23S rRNA^a^ | 23S3 | | CGACCTTCTTCGCCTTAAAGC | 410 | Chu et al., 2008 |
|  |  | 23SA | | TAAGCTGACTAATACTAATTACCC |  |  |
|  | 5S-23S rRNA^a^ | primer 1 | | CTGCGAGTTCGCGGGAGA | 226-266 | Postic et al., 1994 |
|  |  | primer 2 | | TCCTAGGCATTCACCATA |  |  |
| Piroplasmida | 18S rRNA^a^ | PIRO-A | | AATACCCAATCCTGACACAGGG | 408 | Olmeda et al., 1997 |
|  |  | PIRO-B | | TTAAATACGAATGCCCCCAAC |  |  |
| *Coxiella* | 16S rRNA^a^ | Cox sp434F | | CCTTTTGAGCGTTGACGTTA | 940-950 | Lalzar et al., 2012 |
|  |  | Cox sp1004R | | CCAAAGGCACCAAGTCATTT |  |  |
| *Francisella* | *rpo*B^a^ | FRCLF2 | | GAGGCACACTTAGGTTTAGYTTC | 306 | Duron et al., 2017 |
|  |  | FRCLR2 | | CTAAACTATATGARCCAGTYGACC |  |  |
|  |  | FRCLR1 | | GCATATACATATAACCAACTG |  |  |
| *Midichloria* | 16S rRNA^a^ | Midi-F | | GTACATGGGAATCTACCTTGC | 691-675 | Cafiso et al., 2016 |
|  |  | Midi-R | | CAGGTCGCCCTATTGCTTCTTT |  |  |
|  |  | Midi-F2 | | CAAAAGTGAAAGCCTTGGGC |  |  |
|  |  | Midi-R2 | | TGAGACTTAAAYCCCAACATC |  |  |

a PCRs performed to all the samples.

b PCRs performed to selected samples (explained in the main text)

**References**

Beati, L., Keirans, J.E., 2001. Analysis of the systematic relationships among ticks of the genera *Rhipicephalus* and *Boophilus* (Acari: Ixodidae) based on mitochondrial 12S ribosomal DNA gene sequences and morphological characters. J. Parasitol. 87, 32–48. https://doi.org/10.1645/0022-3395(2001)087[0032:AOTSRA]2.0.CO;2.

Cafiso, A., Bazzocchi, C., De Marco, L., Opara, M.N., Sassera, D., Plantard, O., 2016. Molecular screening for *Midichloria* in hard and soft ticks reveals variable prevalence levels and bacterial loads in different tick species. Ticks Tick. Borne. Dis. 7, 1186–1192. https://doi.org/10.1016/j.ttbdis.2016.07.017.

Chu, C.Y., Jiang, B.G., Liu, W., Zhao, Q.M., Wu, X.M., Zhang, P.H., Zhan, L., Yang, H., Cao, W.C., 2008. Presence of pathogenic *Borrelia burgdorferi* sensu lato in ticks and rodents in Zhejiang, south-east China. J. Med. Microbiol. 57, 980–985. https://doi.org/10.1099/jmm.0.47663-0.

Duron, O., Binetruy, F., Noël, V., Cremaschi, J., McCoy, K.D., Arnathau, C., Plantard, O., Goolsby, J., Pérez de León, A.A., Heylen, D.J.A., Van Oosten, A.R., Gottlieb, Y., Baneth, G., Guglielmone, A.A., Estrada-Peña, A., Opara, M.N., Zenner, L., Vavre, F., Chevillon, C., 2017. Evolutionary changes in symbiont community structure in ticks. Mol. Ecol. 26, 2905–2921. https://doi.org/10.1111/mec.14094.

Hornok, S., Földvári, G., Elek, V., Naranjo, V., Farkas, R., de la Fuente, J., 2008. Molecular identification of *Anaplasma marginale* and rickettsial endosymbionts in blood-sucking flies (Diptera: Tabanidae, Muscidae) and hard ticks (Acari: Ixodidae). Vet. Parasitol. 154, 354–359. https://doi.org/10.1016/j.vetpar.2008.03.019.

Kimita, G., Mutai, B., Nyanjom, S.G., Wamunyokoli, F., Waitumbi, J., 2016. Phylogenetic Variants of *Rickettsia africae*, and Incidental Identification of “*Candidatus* Rickettsia Moyalensis” in Kenya. PLoS Negl. Trop. Dis. 10, 1–14. https://doi.org/10.1371/journal.pntd.0004788.

Lalzar, I., Harrus, S., Mumcuoglu, K.Y., Gottlieb, Y., 2012. Composition and seasonal variation of *Rhipicephalus turanicus* and *Rhipicephalus sanguineus* bacterial communities. Appl. Environ. Microbiol. 78, 4110–4116. https://doi.org/10.1128/AEM.00323-12.

Olmeda, A.S., Armstrong, P.M., Rosenthal, B.M., Valladares, B., Del Castillo, A., De Armas, F., Miguelez, M., Gonzalez, A., Rodrıguez, J.A.R., Spielman, A., 1997. A subtropical case of human babesiosis. Acta Trop. 67, 229–234.

Postic, D., Assous, M. V., Grimont, P.A.D., Baranton, G., 1994. Diversity of *Borrelia burgdorferi* sensu lato evidenced by restriction fragment length polymorphism of rrf (5S)-rrl (23S) intergenic spacer amplicons. Int. J. Syst. Bacteriol. 44, 743–752. https://doi.org/10.1099/00207713-44-4-743.

Roux, V., Rydkina, E., Eremeeva, M.E., Raoult, D., 1997. Citrate synthase gene comparison, a new tool for phylogenetic analysis, and its application for the Rickettsiae. Int. J. Syst. Bacteriol. 47, 252–261. https://doi.org/10.1099/00207713-47-2-252.

Teshale, S., Geysen, D., Ameni, G., Dorny, P., Berkvens, D., 2018. Survey of Anaplasma phagocytophilum and Anaplasma sp. “Omatjenne” infection in cattle in Africa with special reference to Ethiopia. Parasites and Vectors 11, 1–10. https://doi.org/10.1186/s13071-018-2633-y.
