# Supplementary material for: Multi‐country investigation of the diversity and associated microorganisms isolated from tick species from domestic animals, wildlife and vegetation in selected african countries
Source: Exp Appl Acarol. 2021 Mar 1;83(3):427–48. doi: 10.1007/s10493-021-00598-3 (PMC7940270; doi:10.1007/s10493-021-00598-3)
Supplement: Supplementary file 2 — Electronic supplementary material 2 (PDF 466 kb) [file 10493_2021_598_MOESM2_ESM.pdf]

Additional file 2: Figures S1-S7. Maximum likelyhood phylogenetic trees of organisms found in association with African ticks: *Rickettsia gltA* gene (**S1a**), *Rickettsia ompA* gene (**S1b**), *Rickettsia ompB* gene (**S1c**), *Anaplasmataceae* SSU rRNA gene (**S2**), piroplasmid SSU rRNA gene (**S3**), *Borrelia* ITS (**S4**), *Coxiella* SSU rRNA gene (**S5**), *Francisella rpoB* gene (**S6**), "*Ca.* Midichloria" SSU rRNA gene (**S7**). Newly obtained sequences are shown in bold. Numbers on branches indicate bootstrap support (values below 70% are not shown). Scale bars stand for estimated proportional sequences divergence.

Figure S1a

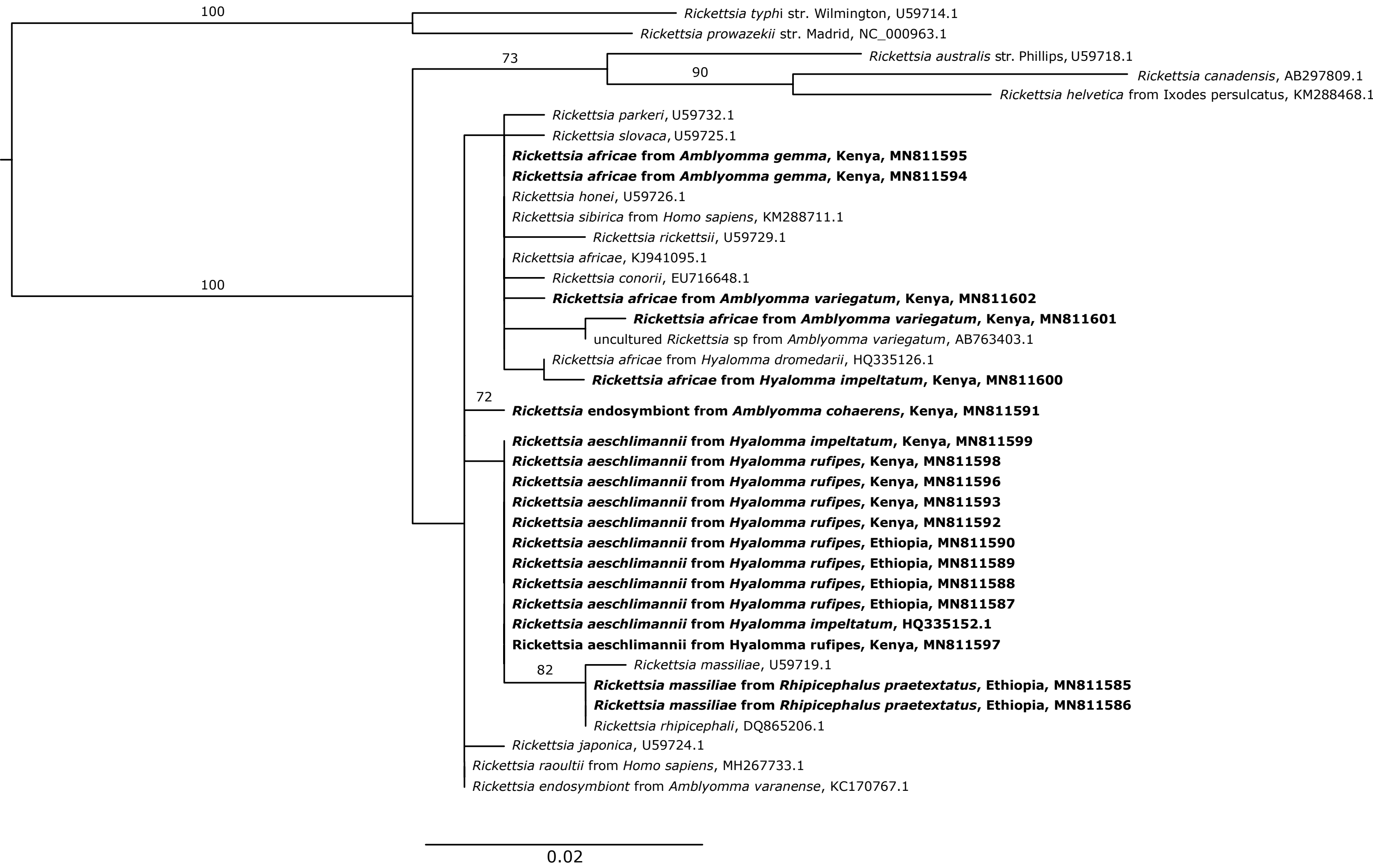
100
Rickettsia typhi str. Wilmington, U59714.1
Rickettsia prowazekii str. Madrid, NC_000963.1
73
Rickettsia australis str. Phillips, U59718.1
90
Rickettsia canadensis, AB297809.1
Rickettsia helvetica from Ixodes persulcatus, KM288468.1
Rickettsia parkeri, U59732.1
Rickettsia slovaca, U59725.1
Rickettsia africae from Amblyomma gemma, Kenya, MN811595
Rickettsia africae from Amblyomma gemma, Kenya, MN811594
Rickettsia honei, U59726.1
Rickettsia sibirica from Homo sapiens, KM288711.1
Rickettsia rickettsii, U59729.1
Rickettsia africae, KJ941095.1
Rickettsia conorii, EU716648.1
100
Rickettsia africae from Amblyomma variegatum, Kenya, MN811602
Rickettsia africae from Amblyomma variegatum, Kenya, MN811601
uncultured Rickettsia sp from Amblyomma variegatum, AB763403.1
Rickettsia africae from Hyalomma dromedarii, HQ335126.1
Rickettsia africae from Hyalomma impeltatum, Kenya, MN811600
72
Rickettsia endosymbiont from Amblyomma cohaerens, Kenya, MN811591
Rickettsia aeschlimannii from Hyalomma impeltatum, Kenya, MN811599
Rickettsia aeschlimannii from Hyalomma rufipes, Kenya, MN811598
Rickettsia aeschlimannii from Hyalomma rufipes, Kenya, MN811596
Rickettsia aeschlimannii from Hyalomma rufipes, Kenya, MN811593
Rickettsia aeschlimannii from Hyalomma rufipes, Kenya, MN811592
Rickettsia aeschlimannii from Hyalomma rufipes, Ethiopia, MN811590
Rickettsia aeschlimannii from Hyalomma rufipes, Ethiopia, MN811589
Rickettsia aeschlimannii from Hyalomma rufipes, Ethiopia, MN811588
Rickettsia aeschlimannii from Hyalomma rufipes, Ethiopia, MN811587
Rickettsia aeschlimannii from Hyalomma impeltatum, HQ335152.1
Rickettsia aeschlimannii from Hyalomma rufipes, Kenya, MN811597
Rickettsia massiliae, U59719.1
82
Rickettsia massiliae from Rhipicephalus praetextatus, Ethiopia, MN811585
Rickettsia massiliae from Rhipicephalus praetextatus, Ethiopia, MN811586
Rickettsia rhipicephali, DQ865206.1
Rickettsia japonica, U59724.1
Rickettsia raoultii from Homo sapiens, MH267733.1
Rickettsia endosymbiont from Amblyomma varanense, KC170767.1
0.02

Figure S1b

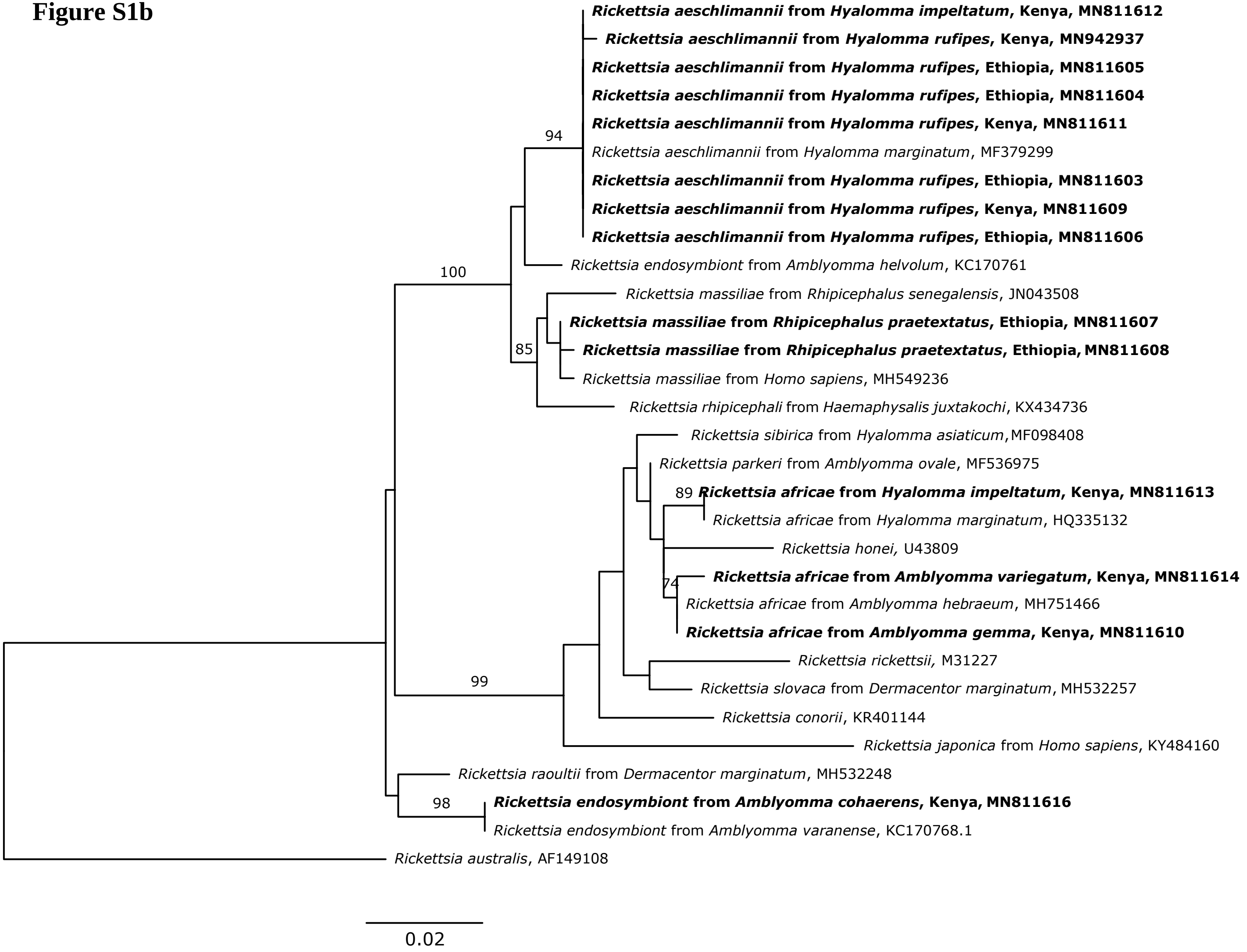

Rickettsia aeschlimannii from Hyalomma impeltatum, Kenya, MN811612
Rickettsia aeschlimannii from Hyalomma rufipes, Kenya, MN942937
Rickettsia aeschlimannii from Hyalomma rufipes, Ethiopia, MN811605
Rickettsia aeschlimannii from Hyalomma rufipes, Ethiopia, MN811604
Rickettsia aeschlimannii from Hyalomma rufipes, Kenya, MN811611
Rickettsia aeschlimannii from Hyalomma marginatum, MF379299
Rickettsia aeschlimannii from Hyalomma rufipes, Ethiopia, MN811603
Rickettsia aeschlimannii from Hyalomma rufipes, Kenya, MN811609
Rickettsia aeschlimannii from Hyalomma rufipes, Ethiopia, MN811606
Rickettsia endosymbiont from Amblyomma helvolum, KC170761
Rickettsia massiliae from Rhipicephalus senegalensis, JN043508
Rickettsia massiliae from Rhipicephalus praetextatus, Ethiopia, MN811607
Rickettsia massiliae from Rhipicephalus praetextatus, Ethiopia, MN811608
Rickettsia massiliae from Homo sapiens, MH549236
Rickettsia rhipicephali from Haemaphysalis juxtakochi, KX434736
Rickettsia sibirica from Hyalomma asiaticum,MF098408
Rickettsia parkeri from Amblyomma ovale, MF536975
Rickettsia africae from Hyalomma impeltatum, Kenya, MN811613
Rickettsia africae from Hyalomma marginatum, HQ335132
Rickettsia honei, U43809
Rickettsia africae from Amblyomma variegatum, Kenya, MN811614
Rickettsia africae from Amblyomma hebraeum, MH751466
Rickettsia africae from Amblyomma gemma, Kenya, MN811610
Rickettsia rickettsii, M31227
Rickettsia slovaca from Dermacentor marginatum, MH532257
Rickettsia conorii, KR401144
Rickettsia japonica from Homo sapiens, KY484160
Rickettsia raoultii from Dermacentor marginatum, MH532248
Rickettsia endosymbiont from Amblyomma cohaerens, Kenya, MN811616
Rickettsia endosymbiont from Amblyomma varanense, KC170768.1
Rickettsia australis, AF149108
94
100
85
89
74
99
98
0.02

Figure S1c

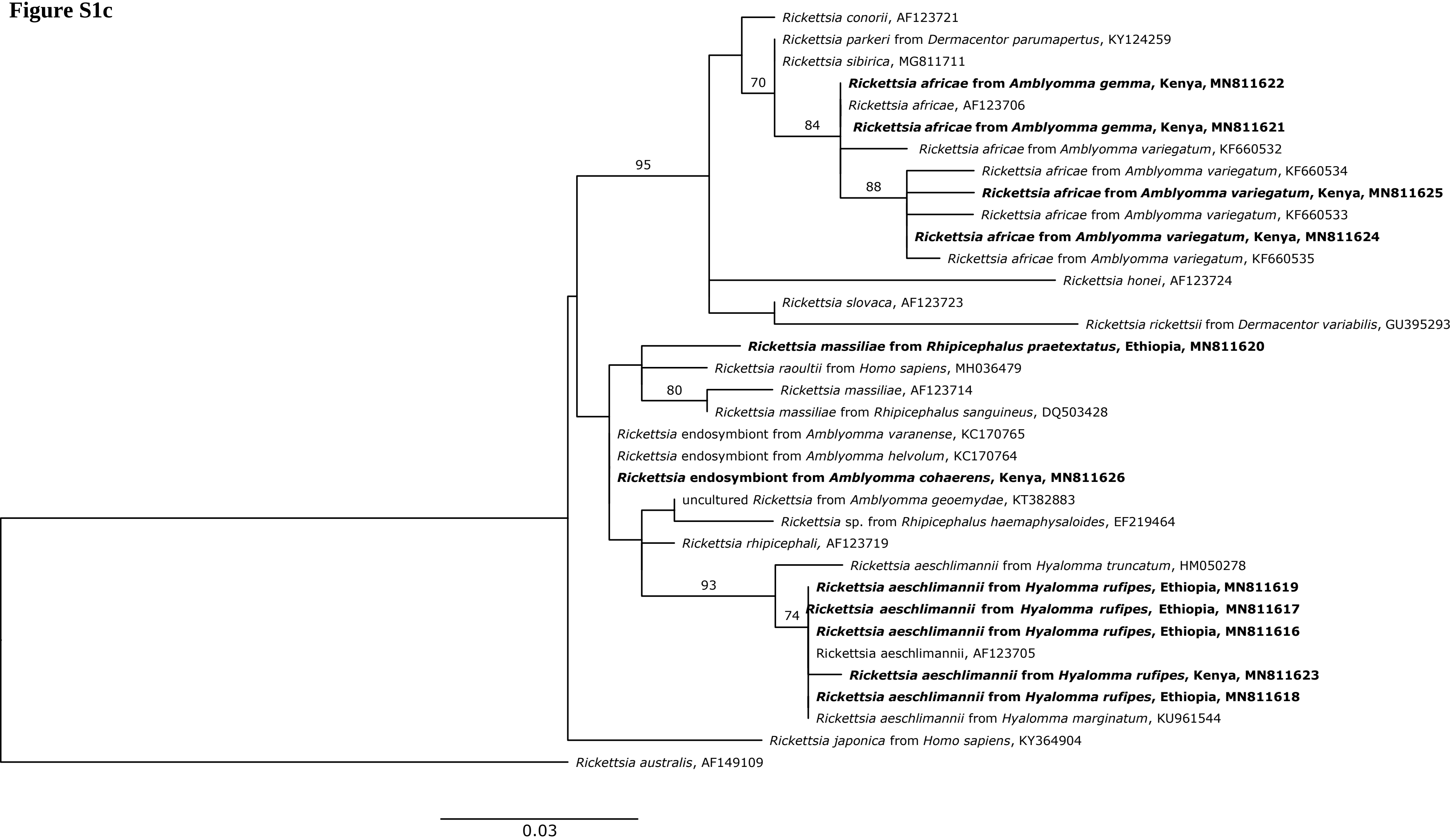

Rickettsia conorii, AF123721
Rickettsia parkeri from Dermacentor parumapertus, KY124259
Rickettsia sibirica, MG811711
70
Rickettsia africae from Amblyomma gemma, Kenya, MN811622
Rickettsia africae, AF123706
84
Rickettsia africae from Amblyomma gemma, Kenya, MN811621
Rickettsia africae from Amblyomma variegatum, KF660532
95
Rickettsia africae from Amblyomma variegatum, KF660534
88
Rickettsia africae from Amblyomma variegatum, Kenya, MN811625
Rickettsia africae from Amblyomma variegatum, KF660533
Rickettsia africae from Amblyomma variegatum, Kenya, MN811624
Rickettsia africae from Amblyomma variegatum, KF660535
Rickettsia honei, AF123724
Rickettsia slovaca, AF123723
Rickettsia rickettsii from Dermacentor variabilis, GU395293
Rickettsia massiliae from Rhipicephalus praetextatus, Ethiopia, MN811620
Rickettsia raoultii from Homo sapiens, MH036479
80
Rickettsia massiliae, AF123714
Rickettsia massiliae from Rhipicephalus sanguineus, DQ503428
Rickettsia endosymbiont from Amblyomma varanense, KC170765
Rickettsia endosymbiont from Amblyomma helvolum, KC170764
Rickettsia endosymbiont from Amblyomma cohaerens, Kenya, MN811626
uncultured Rickettsia from Amblyomma geoemydae, KT382883
Rickettsia sp. from Rhipicephalus haemaphysaloides, EF219464
Rickettsia rhipicephali, AF123719
Rickettsia aeschlimannii from Hyalomma truncatum, HM050278
93
Rickettsia aeschlimannii from Hyalomma rufipes, Ethiopia, MN811619
Rickettsia aeschlimannii from Hyalomma rufipes, Ethiopia, MN811617
74
Rickettsia aeschlimannii from Hyalomma rufipes, Ethiopia, MN811616
Rickettsia aeschlimannii, AF123705
Rickettsia aeschlimannii from Hyalomma rufipes, Kenya, MN811623
Rickettsia aeschlimannii from Hyalomma rufipes, Ethiopia, MN811618
Rickettsia aeschlimannii from Hyalomma marginatum, KU961544
Rickettsia japonica from Homo sapiens, KY364904
Rickettsia australis, AF149109
0.03

Figure S2

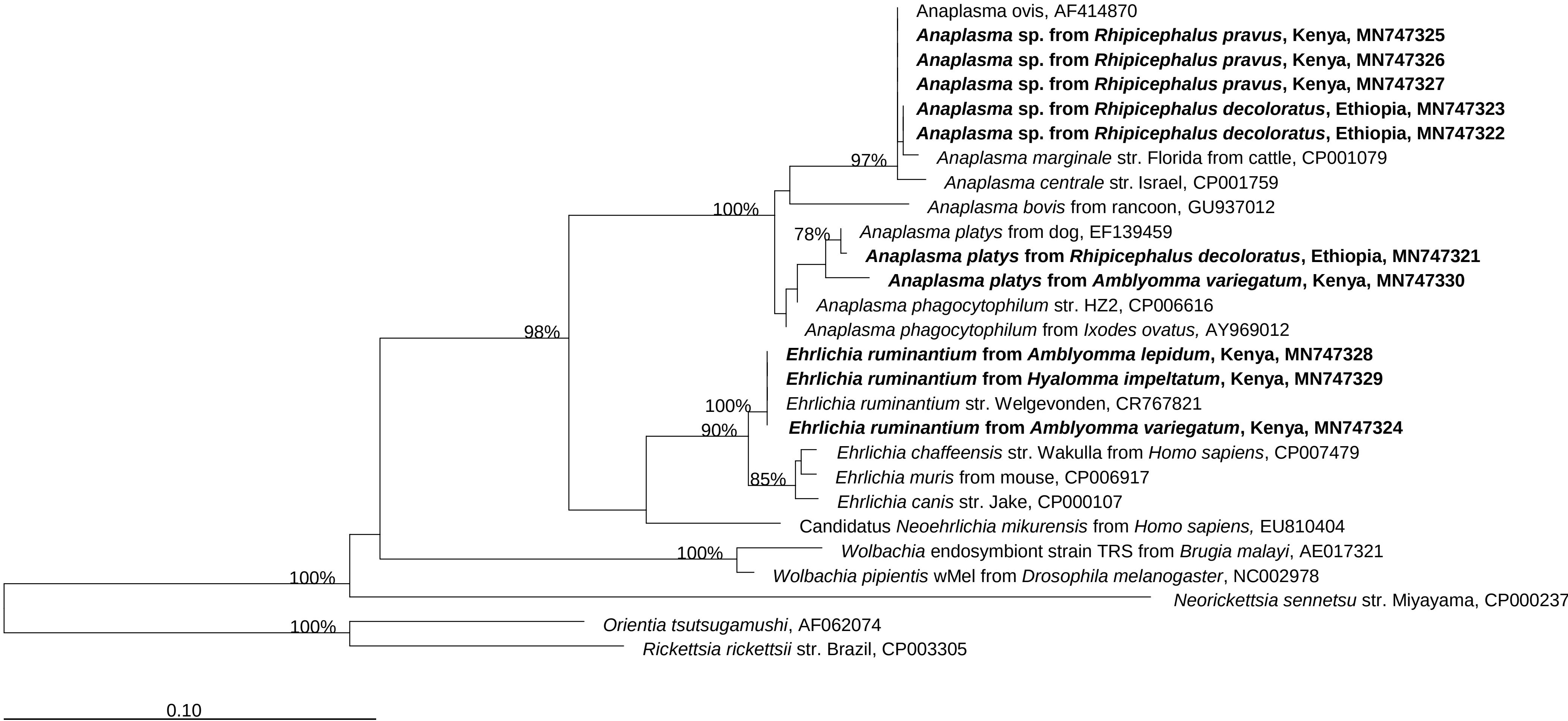
Anaplasma ovis, AF414870
Anaplasma sp. from Rhipicephalus pravus, Kenya, MN747325
Anaplasma sp. from Rhipicephalus pravus, Kenya, MN747326
Anaplasma sp. from Rhipicephalus pravus, Kenya, MN747327
Anaplasma sp. from Rhipicephalus decoloratus, Ethiopia, MN747323
Anaplasma sp. from Rhipicephalus decoloratus, Ethiopia, MN747322
97%
Anaplasma marginale str. Florida from cattle, CP001079
Anaplasma centrale str. Israel, CP001759
100%
Anaplasma bovis from rancoon, GU937012
78%
Anaplasma platys from dog, EF139459
Anaplasma platys from Rhipicephalus decoloratus, Ethiopia, MN747321
Anaplasma platys from Amblyomma variegatum, Kenya, MN747330
Anaplasma phagocytophilum str. HZ2, CP006616
98%
Anaplasma phagocytophilum from Ixodes ovatus, AY969012
Ehrlichia ruminantium from Amblyomma lepidum, Kenya, MN747328
Ehrlichia ruminantium from Hyalomma impeltatum, Kenya, MN747329
100%
Ehrlichia ruminantium str. Welgevonden, CR767821
90%
Ehrlichia ruminantium from Amblyomma variegatum, Kenya, MN747324
Ehrlichia chaffeensis str. Wakulla from Homo sapiens, CP007479
85%
Ehrlichia muris from mouse, CP006917
Ehrlichia canis str. Jake, CP000107
Candidatus Neoehrlichia mikurensis from Homo sapiens, EU810404
100%
Wolbachia endosymbiont strain TRS from Brugia malayi, AE017321
100%
Wolbachia pipientis wMel from Drosophila melanogaster, NC002978
Neorickettsia sennetsu str. Miyayama, CP000237
100%
Orientia tsutsugamushi, AF062074
Rickettsia rickettsii str. Brazil, CP003305
0.10

Figure S3

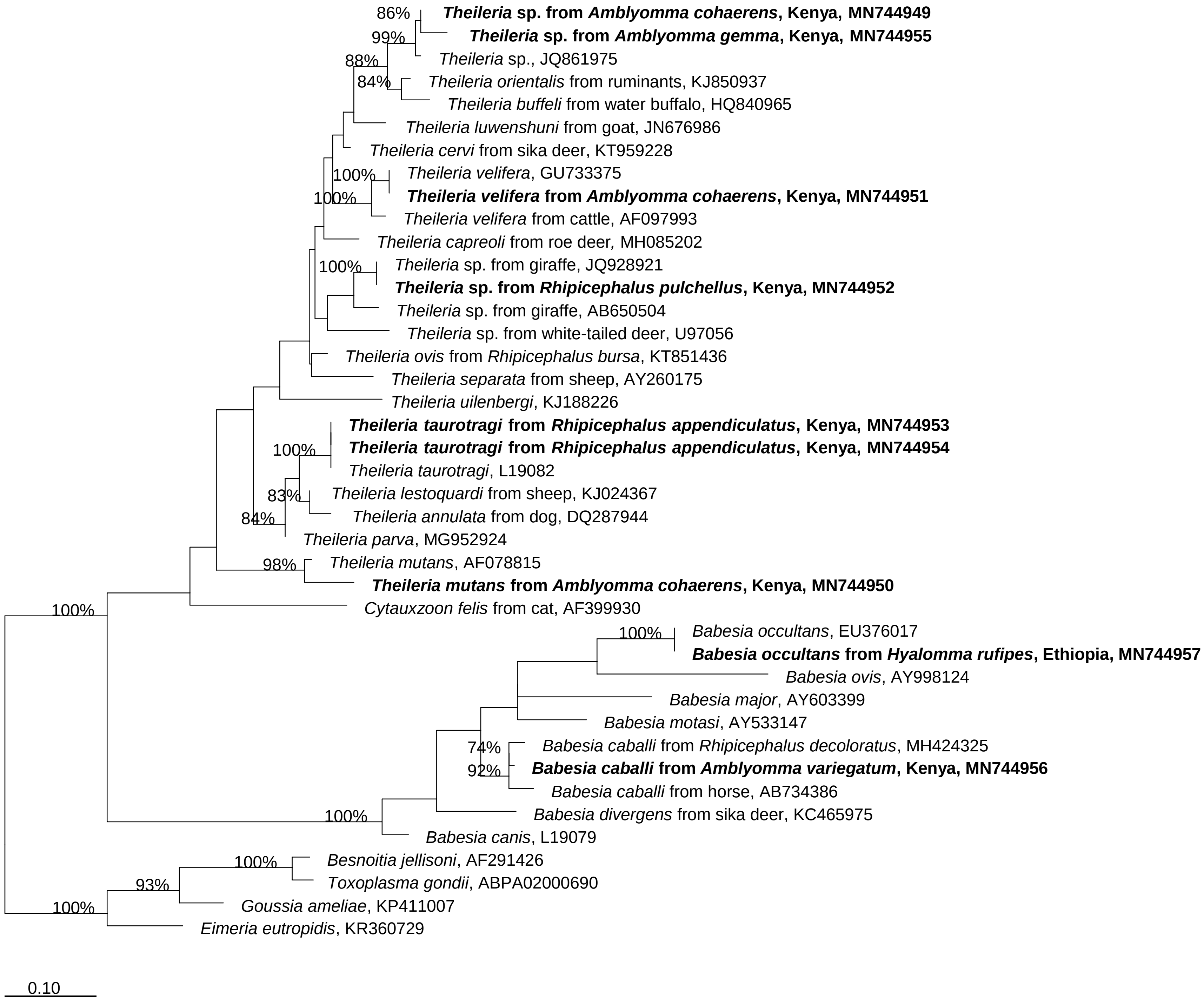
86%
Theileria sp. from Amblyomma cohaerens, Kenya, MN744949
99%
Theileria sp. from Amblyomma gemma, Kenya, MN744955
88%
Theileria sp., JQ861975
84%
Theileria orientalis from ruminants, KJ850937
Theileria buffeli from water buffalo, HQ840965
Theileria luwenshuni from goat, JN676986
Theileria cervi from sika deer, KT959228
100%
Theileria velifera, GU733375
100%
Theileria velifera from Amblyomma cohaerens, Kenya, MN744951
Theileria velifera from cattle, AF097993
Theileria capreoli from roe deer, MH085202
100%
Theileria sp. from giraffe, JQ928921
Theileria sp. from Rhipicephalus pulchellus, Kenya, MN744952
Theileria sp. from giraffe, AB650504
Theileria sp. from white-tailed deer, U97056
Theileria ovis from Rhipicephalus bursa, KT851436
Theileria separata from sheep, AY260175
Theileria uilenbergi, KJ188226
Theileria taurotragi from Rhipicephalus appendiculatus, Kenya, MN744953
100%
Theileria taurotragi from Rhipicephalus appendiculatus, Kenya, MN744954
Theileria taurotragi, L19082
83%
Theileria lestoquardi from sheep, KJ024367
84%
Theileria annulata from dog, DQ287944
Theileria parva, MG952924
98%
Theileria mutans, AF078815
Theileria mutans from Amblyomma cohaerens, Kenya, MN744950
100%
Cytauxzoon felis from cat, AF399930
100%
Babesia occultans, EU376017
Babesia occultans from Hyalomma rufipes, Ethiopia, MN744957
Babesia ovis, AY998124
Babesia major, AY603399
Babesia motasi, AY533147
74%
Babesia caballi from Rhipicephalus decoloratus, MH424325
92%
Babesia caballi from Amblyomma variegatum, Kenya, MN744956
Babesia caballi from horse, AB734386
100%
Babesia divergens from sika deer, KC465975
Babesia canis, L19079
100%
Besnoitia jellisoni, AF291426
93%
Toxoplasma gondii, ABPA02000690
100%
Goussia ameliae, KP411007
Eimeria eutropidis, KR360729
0.10

Figure S4

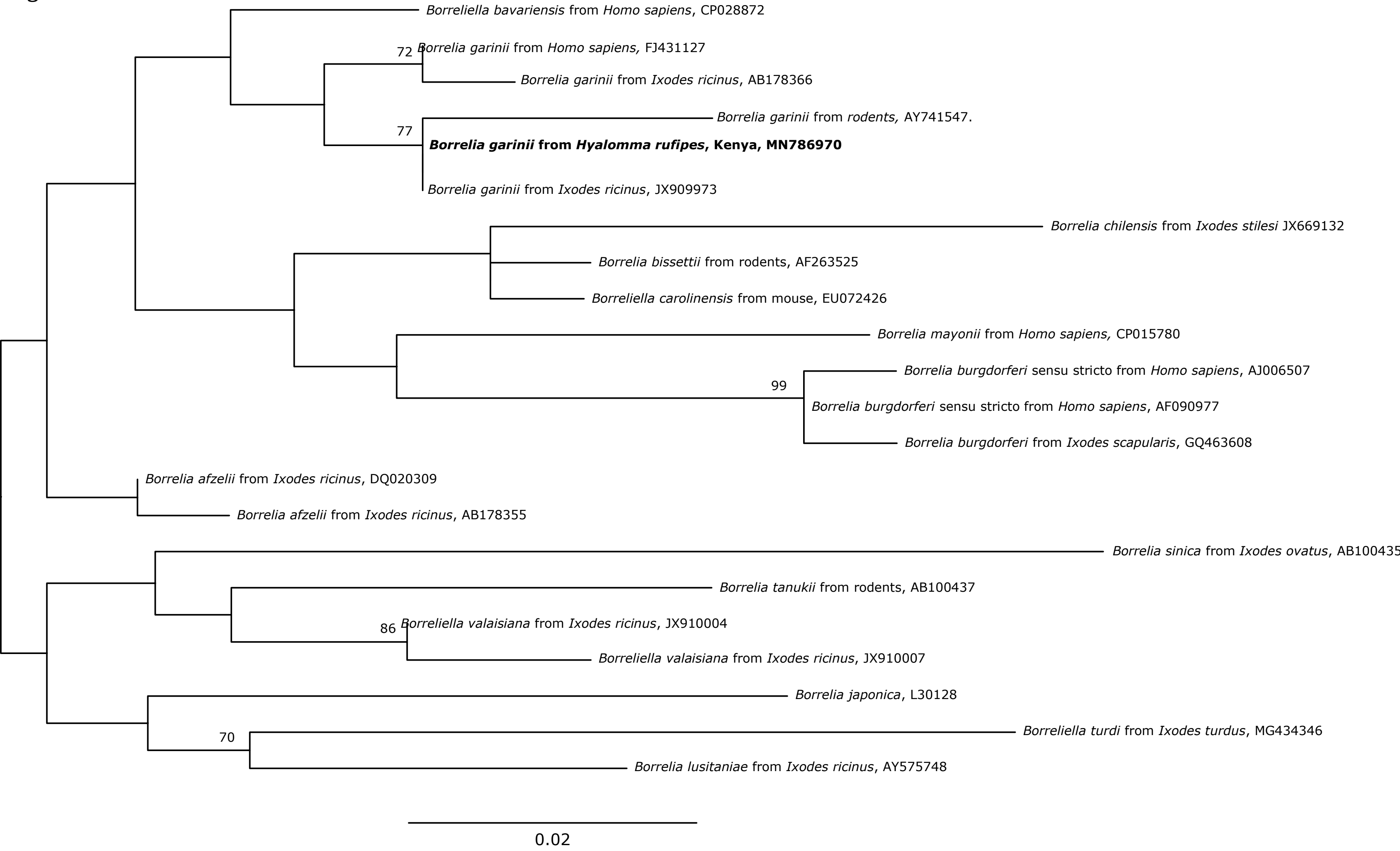
Borreliella bavariensis from Homo sapiens, CP028872
72
Borrelia garinii from Homo sapiens, FJ431127
Borrelia garinii from Ixodes ricinus, AB178366
Borrelia garinii from rodents, AY741547.
77
Borrelia garinii from Hyalomma rufipes, Kenya, MN786970
Borrelia garinii from Ixodes ricinus, JX909973
Borrelia chilensis from Ixodes stilesi JX669132
Borrelia bissettii from rodents, AF263525
Borreliella carolinensis from mouse, EU072426
Borrelia mayonii from Homo sapiens, CP015780
99
Borrelia burgdorferi sensu stricto from Homo sapiens, AJ006507
Borrelia burgdorferi sensu stricto from Homo sapiens, AF090977
Borrelia burgdorferi from Ixodes scapularis, GQ463608
Borrelia afzelii from Ixodes ricinus, DQ020309
Borrelia afzelii from Ixodes ricinus, AB178355
Borrelia sinica from Ixodes ovatus, AB100435
Borrelia tanukii from rodents, AB100437
86
Borreliella valaisiana from Ixodes ricinus, JX910004
Borreliella valaisiana from Ixodes ricinus, JX910007
Borrelia japonica, L30128
70
Borreliella turdi from Ixodes turdus, MG434346
Borrelia lusitaniae from Ixodes ricinus, AY575748
0.02

Figure S5

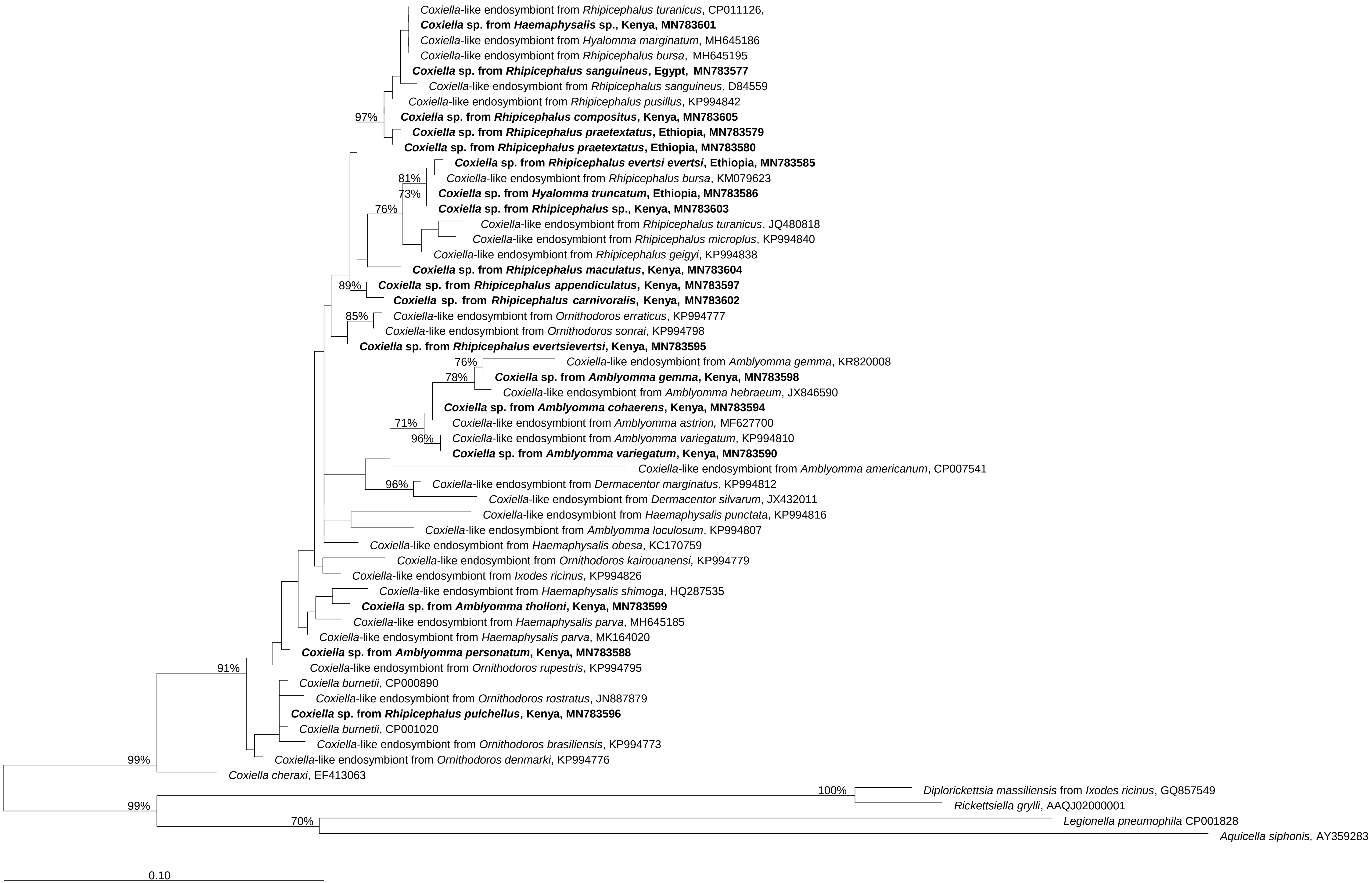
Coxiella-like endosymbiont from Rhipicephalus turanicus, CP011126,
Coxiella sp. from Haemaphysalis sp., Kenya, MN783601
Coxiella-like endosymbiont from Hyalomma marginatum, MH645186
Coxiella-like endosymbiont from Rhipicephalus bursa, MH645195
Coxiella sp. from Rhipicephalus sanguineus, Egypt, MN783577
Coxiella-like endosymbiont from Rhipicephalus sanguineus, D84559
Coxiella-like endosymbiont from Rhipicephalus pusillus, KP994842
97%
Coxiella sp. from Rhipicephalus compositus, Kenya, MN783605
Coxiella sp. from Rhipicephalus praetextatus, Ethiopia, MN783579
Coxiella sp. from Rhipicephalus praetextatus, Ethiopia, MN783580
Coxiella sp. from Rhipicephalus evertsi evertsi, Ethiopia, MN783585
81%
Coxiella-like endosymbiont from Rhipicephalus bursa, KM079623
73%
Coxiella sp. from Hyalomma truncatum, Ethiopia, MN783586
76%
Coxiella sp. from Rhipicephalus sp., Kenya, MN783603
Coxiella-like endosymbiont from Rhipicephalus turanicus, JQ480818
Coxiella-like endosymbiont from Rhipicephalus microplus, KP994840
Coxiella-like endosymbiont from Rhipicephalus geigyi, KP994838
Coxiella sp. from Rhipicephalus maculatus, Kenya, MN783604
89%
Coxiella sp. from Rhipicephalus appendiculatus, Kenya, MN783597
Coxiella sp. from Rhipicephalus carnivoralis, Kenya, MN783602
85%
Coxiella-like endosymbiont from Ornithodoros erraticus, KP994777
Coxiella-like endosymbiont from Ornithodoros sonrai, KP994798
Coxiella sp. from Rhipicephalus evertsievertsi, Kenya, MN783595
76%
Coxiella-like endosymbiont from Amblyomma gemma, KR820008
78%
Coxiella sp. from Amblyomma gemma, Kenya, MN783598
Coxiella-like endosymbiont from Amblyomma hebraeum, JX846590
Coxiella sp. from Amblyomma cohaerens, Kenya, MN783594
71%
Coxiella-like endosymbiont from Amblyomma astrion, MF627700
96%
Coxiella-like endosymbiont from Amblyomma variegatum, KP994810
Coxiella sp. from Amblyomma variegatum, Kenya, MN783590
Coxiella-like endosymbiont from Amblyomma americanum, CP007541
96%
Coxiella-like endosymbiont from Dermacentor marginatus, KP994812
Coxiella-like endosymbiont from Dermacentor silvarum, JX432011
Coxiella-like endosymbiont from Haemaphysalis punctata, KP994816
Coxiella-like endosymbiont from Amblyomma loculosum, KP994807
Coxiella-like endosymbiont from Haemaphysalis obesa, KC170759
Coxiella-like endosymbiont from Ornithodoros kairouanensi, KP994779
Coxiella-like endosymbiont from Ixodes ricinus, KP994826
Coxiella-like endosymbiont from Haemaphysalis shimoga, HQ287535
Coxiella sp. from Amblyomma tholloni, Kenya, MN783599
Coxiella-like endosymbiont from Haemaphysalis parva, MH645185
Coxiella-like endosymbiont from Haemaphysalis parva, MK164020
Coxiella sp. from Amblyomma personatum, Kenya, MN783588
91%
Coxiella-like endosymbiont from Ornithodoros rupestris, KP994795
Coxiella burnetii, CP000890
Coxiella-like endosymbiont from Ornithodoros rostratus, JN887879
Coxiella sp. from Rhipicephalus pulchellus, Kenya, MN783596
Coxiella burnetii, CP001020
Coxiella-like endosymbiont from Ornithodoros brasiliensis, KP994773
99%
Coxiella-like endosymbiont from Ornithodoros denmarki, KP994776
Coxiella cheraxi, EF413063
100%
Diplorickettsia massiliensis from Ixodes ricinus, GQ857549
99%
Rickettsiella grylli, AAQJ02000001
70%
Legionella pneumophila CP001828
Aquicella siphonis, AY359283
0.10

Figure S6

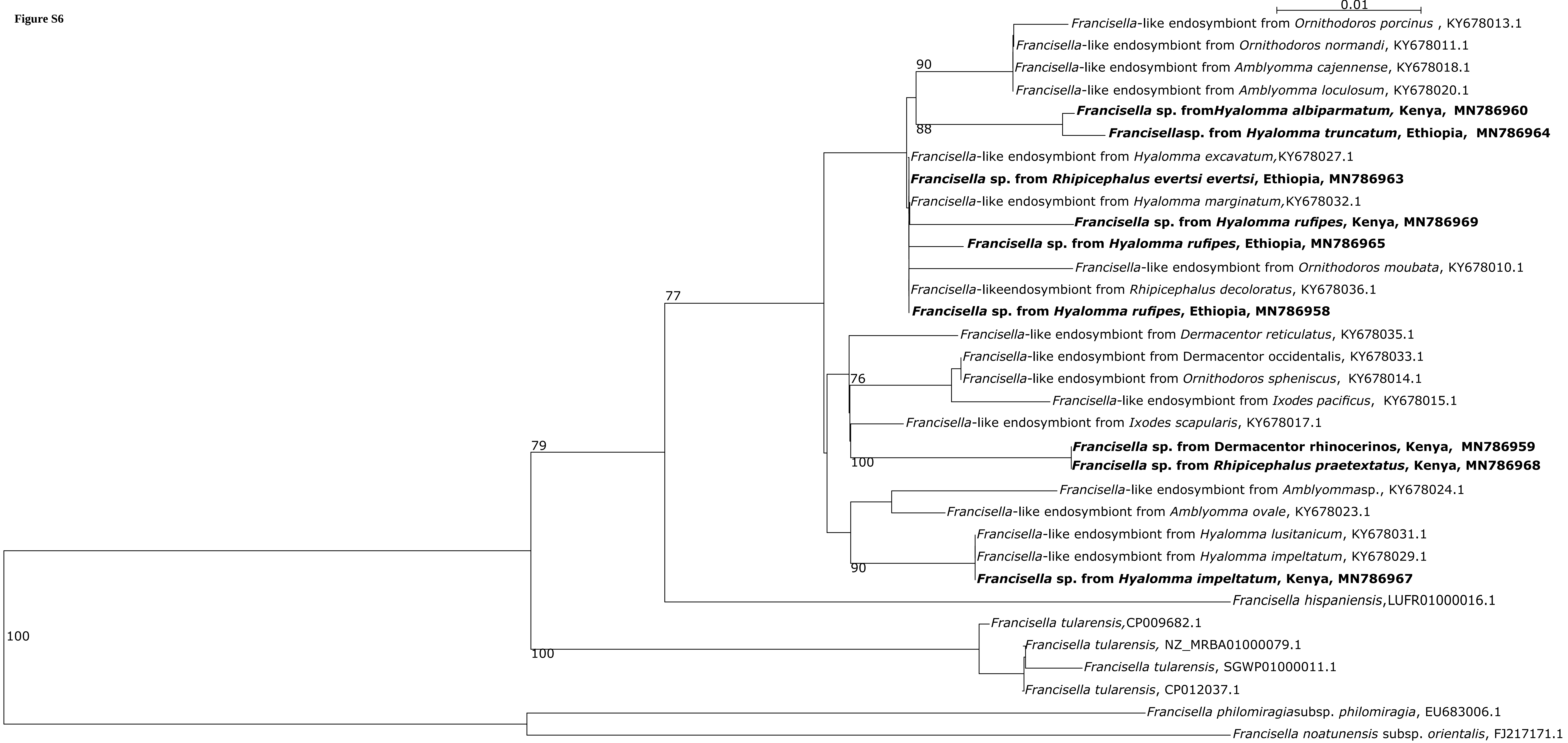
0.01
Francisella-like endosymbiont from Ornithodoros porcinus , KY678013.1
Francisella-like endosymbiont from Ornithodoros normandi, KY678011.1
Francisella-like endosymbiont from Amblyomma cajennense, KY678018.1
Francisella-like endosymbiont from Amblyomma loculosum, KY678020.1
Francisella sp. fromHyalomma albiparmatum, Kenya, MN786960
Francisellasp. from Hyalomma truncatum, Ethiopia, MN786964
Francisella-like endosymbiont from Hyalomma excavatum,KY678027.1
Francisella sp. from Rhipicephalus evertsi evertsi, Ethiopia, MN786963
Francisella-like endosymbiont from Hyalomma marginatum,KY678032.1
Francisella sp. from Hyalomma rufipes, Kenya, MN786969
Francisella sp. from Hyalomma rufipes, Ethiopia, MN786965
Francisella-like endosymbiont from Ornithodoros moubata, KY678010.1
Francisella-likeendosymbiont from Rhipicephalus decoloratus, KY678036.1
Francisella sp. from Hyalomma rufipes, Ethiopia, MN786958
Francisella-like endosymbiont from Dermacentor reticulatus, KY678035.1
Francisella-like endosymbiont from Dermacentor occidentalis, KY678033.1
Francisella-like endosymbiont from Ornithodoros spheniscus, KY678014.1
Francisella-like endosymbiont from Ixodes pacificus, KY678015.1
Francisella-like endosymbiont from Ixodes scapularis, KY678017.1
Francisella sp. from Dermacentor rhinocerinos, Kenya, MN786959
Francisella sp. from Rhipicephalus praetextatus, Kenya, MN786968
Francisella-like endosymbiont from Amblyommasp., KY678024.1
Francisella-like endosymbiont from Amblyomma ovale, KY678023.1
Francisella-like endosymbiont from Hyalomma lusitanicum, KY678031.1
Francisella-like endosymbiont from Hyalomma impeltatum, KY678029.1
Francisella sp. from Hyalomma impeltatum, Kenya, MN786967
Francisella hispaniensis,LUFR01000016.1
Francisella tularensis,CP009682.1
Francisella tularensis, NZ_MRBA01000079.1
Francisella tularensis, SGWP01000011.1
Francisella tularensis, CP012037.1
Francisella philomiragiasubsp. philomiragia, EU683006.1
Francisella noatunensis subsp. orientalis, FJ217171.1
90
88
77
76
100
90
79
100
100

Figure S7

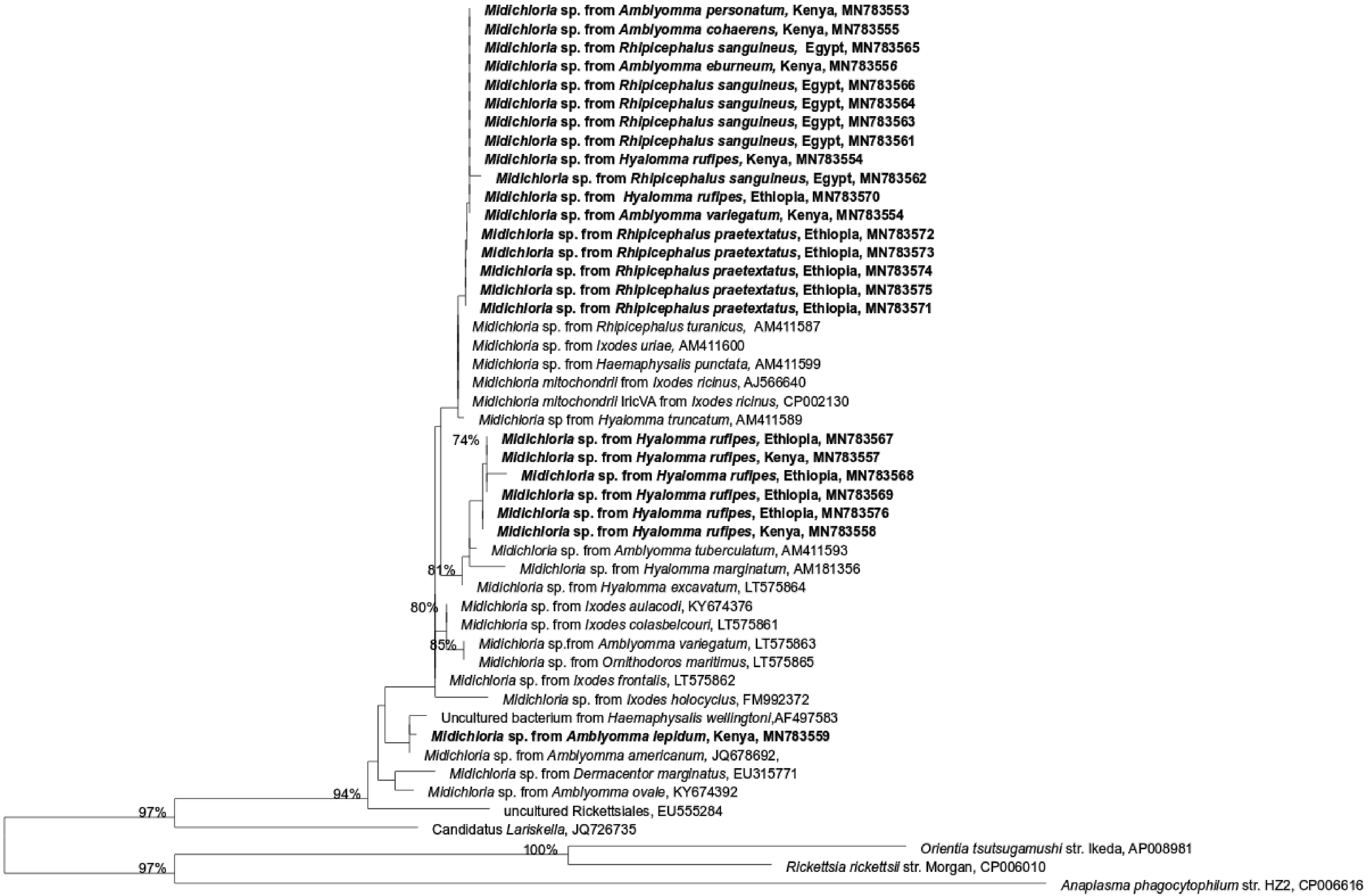

Midichloria sp. from Amblyomma personatum, Kenya, MN783553
Midichloria sp. from Amblyomma cohaerens, Kenya, MN783555
Midichloria sp. from Rhipicephalus sanguineus, Egypt, MN783565
Midichloria sp. from Amblyomma eburneum, Kenya, MN783556
Midichloria sp. from Rhipicephalus sanguineus, Egypt, MN783566
Midichloria sp. from Rhipicephalus sanguineus, Egypt, MN783564
Midichloria sp. from Rhipicephalus sanguineus, Egypt, MN783563
Midichloria sp. from Rhipicephalus sanguineus, Egypt, MN783561
Midichloria sp. from Hyalomma rufipes, Kenya, MN783554
Midichloria sp. from Rhipicephalus sanguineus, Egypt, MN783562
Midichloria sp. from Hyalomma rufipes, Ethiopia, MN783570
Midichloria sp. from Amblyomma variegatum, Kenya, MN783554
Midichloria sp. from Rhipicephalus praetextatus, Ethiopia, MN783572
Midichloria sp. from Rhipicephalus praetextatus, Ethiopia, MN783573
Midichloria sp. from Rhipicephalus praetextatus, Ethiopia, MN783574
Midichloria sp. from Rhipicephalus praetextatus, Ethiopia, MN783575
Midichloria sp. from Rhipicephalus praetextatus, Ethiopia, MN783571
Midichloria sp. from Rhipicephalus turanicus, AM411587
Midichloria sp. from Ixodes uriae, AM411600
Midichloria sp. from Haemaphysalis punctata, AM411599
Midichloria mitochondrii from Ixodes ricinus, AJ566640
Midichloria mitochondrii IricVA from Ixodes ricinus, CP002130
Midichloria sp from Hyalomma truncatum, AM411589
74%
Midichloria sp. from Hyalomma rufipes, Ethiopia, MN783567
Midichloria sp. from Hyalomma rufipes, Kenya, MN783557
Midichloria sp. from Hyalomma rufipes, Ethiopia, MN783568
Midichloria sp. from Hyalomma rufipes, Ethiopia, MN783569
Midichloria sp. from Hyalomma rufipes, Ethiopia, MN783576
Midichloria sp. from Hyalomma rufipes, Kenya, MN783558
Midichloria sp. from Amblyomma tuberculatum, AM411593
81%
Midichloria sp. from Hyalomma marginatum, AM181356
Midichloria sp. from Hyalomma excavatum, LT575864
80%
Midichloria sp. from Ixodes aulacodi, KY674376
Midichloria sp. from Ixodes colasbelcouri, LT575861
85%
Midichloria sp.from Amblyomma variegatum, LT575863
Midichloria sp. from Ornithodoros maritimus, LT575865
Midichloria sp. from Ixodes frontalis, LT575862
Midichloria sp. from Ixodes holocyclus, FM992372
Uncultured bacterium from Haemaphysalis wellingtoni,AF497583
Midichloria sp. from Amblyomma lepidum, Kenya, MN783559
Midichloria sp. from Amblyomma americanum, JQ678692,
Midichloria sp. from Dermacentor marginatus, EU315771
94%
Midichloria sp. from Amblyomma ovale, KY674392
97%
uncultured Rickettsiales, EU555284
Candidatus Lariskella, JQ726735
100%
Orientia tsutsugamushi str. Ikeda, AP008981
97%
Rickettsia rickettsii str. Morgan, CP006010
Anaplasma phagocytophilum str. HZ2, CP006616
