# Supplementary material for: Multi‐country investigation of the diversity and associated microorganisms isolated from tick species from domestic animals, wildlife and vegetation in selected african countries
Source: Exp Appl Acarol. 2021 Mar 1;83(3):427–48. doi: 10.1007/s10493-021-00598-3 (PMC7940270; doi:10.1007/s10493-021-00598-3)

**Additional file 3: Fig. S8** Co-presence of microorganisms resulted to be statistically significant. Histograms show the null distribution obtained through 9,999 permutations of the observed presence/absence matrixes. A) *Rickettsia* - *Francisella*; B) *Coxiella* - *Francisella*. Blue dashed lines correspond to 2.5^th^ and 97.5^th^ percentiles, red lines represent the observed values.


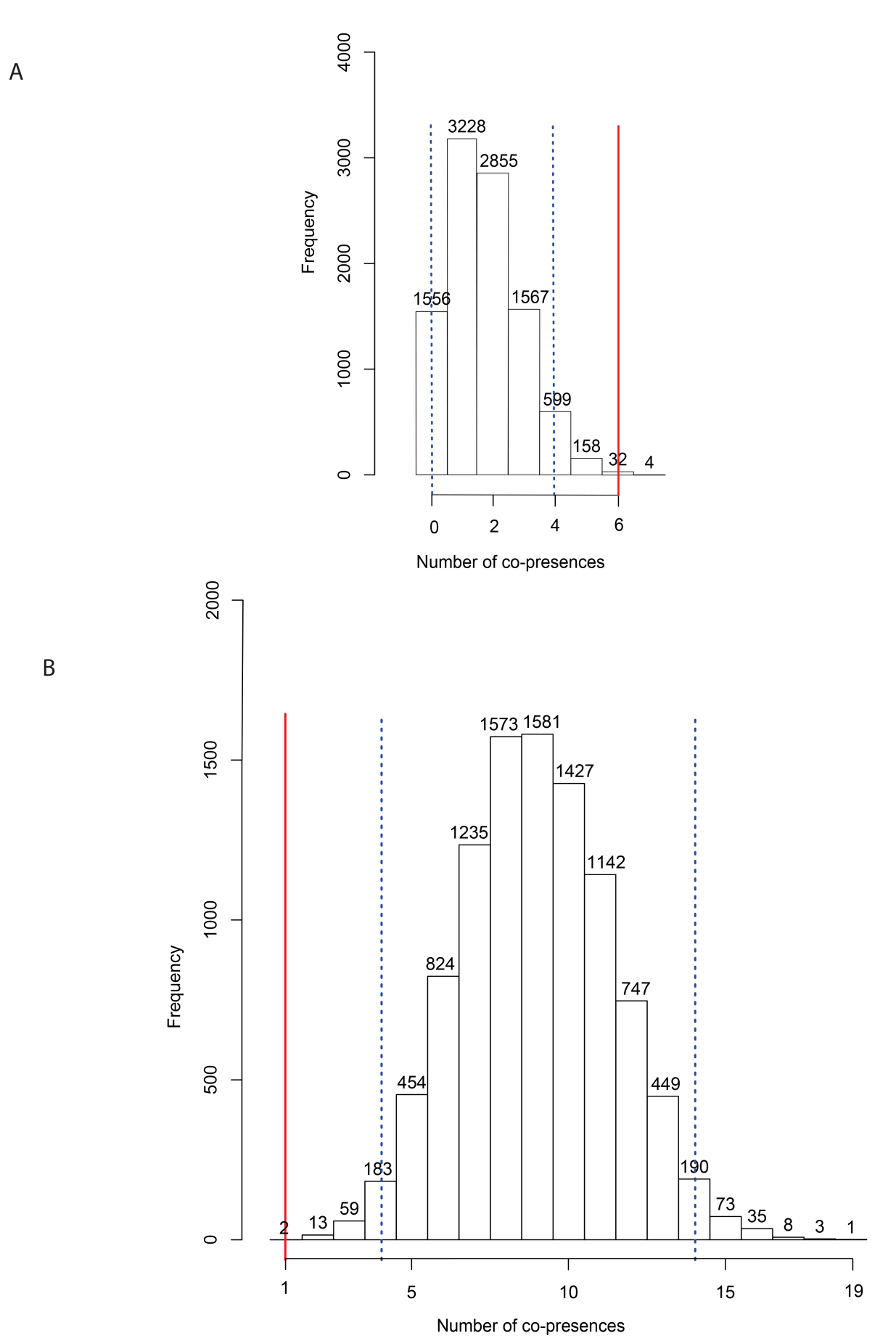

Supplement: Supplementary file 3 — Electronic supplementary material 3 (DOCX 170 kb) [file 10493_2021_598_MOESM3_ESM.docx]
